# Multianalytical Study of Amuletic and Talismanic Islamic‐African Paper Manuscripts in the Slovene Ethnographic Museum

**DOI:** 10.1002/cplu.202500433

**Published:** 2025-11-20

**Authors:** Abdelrazek Elnaggar, Hend Mahgoub, Laura Maestro‐Guijarro, Ana Crespo Ibáñez, Paula María Carmona‐Quiroga, Santiago Sánchez‐Cortés, Žiga Rehar, Gregor Kos, Ahmed Ameen, Marko Frelih, Matija Strlič, Mohamed Oujja, Marta Castillejo

**Affiliations:** ^1^ Heritage Science Lab Ljubljana Faculty of Chemistry and Chemical Technology University of Ljubljana Ljubljana Slovenia; ^2^ Archaeological Science and Excavation Department Faculty of Archaeology Ain Shams University Cairo Egypt; ^3^ Instituto de Química Física Blas Cabrera IQF‐CSIC Madrid Spain; ^4^ Instituto de Estructura de la Materia IEM‐CSIC Madrid Spain; ^5^ The Slovene Ethnographic Museum (SEM) Ljubljana Slovenia; ^6^ College of Arts, Humanities, and Social Sciences University of Sharjah Sharjah UAE; ^7^ UCL Institute for Sustainable Heritage University College London London UK

**Keywords:** fiber furnish analysis, hyperspectral imaging, iron gall ink, Islamic papermaking, organic dyes, paper and ink analysis, Raman spectroscopy, spectroscopic techniques

## Abstract

In contrast to its European counterpart, Islamic papermaking is still little researched, especially in scientific and conservation contexts. This study presents the first in‐depth material analysis of a unique collection of Islamic‐African amulets and talismans from the nineteenth and twentieth centuries, held at the Slovene Ethnographic Museum. This research employed a multi‐analytical approach that included pH measurements, analysis of fibrous materials, iodine test for the presence of starch, hyperspectral imaging (HSI), FTIR‐ATR, Raman spectroscopy, laser‐induced fluorescence (LIF), and laser‐induced breakdown spectroscopy (LIBS), as well as cultural interpretations. Twelve selected manuscripts were examined to characterize paper, inks, dyes, and calligraphic features. The results showed the use of iron gall inks, plant‐based dyes, and mixed paper fibers (straw and softwood pulp), suggesting a mixture of local and imported materials from the colonial period. The calligraphic and decorative styles reflect a synthesis of orthodox Qur’an and local West African Sufi traditions, often incorporating protective texts, magic squares, and regional variants of Kufic script. The findings shed light on technological aspects of Islamic manuscript production in West Africa and support the informed conservation, display, and interpretation of these culturally and spiritually significant objects. This research sets a precedent for comparative heritage studies and enhances the understanding of Islamic material culture in African contexts.

## Graphical abstract

The image illustrates a multi‐technique analysis of Islamic‐African manuscripts from the nineteenth to twentieth centuries. It highlights methods like microscopy, hyperspectral imaging, FTIR, LIF, Raman, LIBS, and furnish analysis used to study paper fibers, inks, and dyes—revealing a blend of local and trade materials and a rich fusion of Qur’anic and West African traditions.

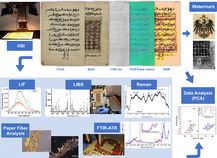

## 1 Introduction

Amulets and talismans from the Islamic world have long fascinated collectors and scholars from the intersection of philology, historical anthropology, religious studies, and the study of material culture [1]. Whether private or public, these collections were collected under specific historical circumstances (e.g., European colonial collectorship, for esthetic reasons, as magical objects, and for folkloric interest) with articulated narratives, that have influenced the way they have been studied and understood. Amulets and talismans are often brought together to a museum or a library as part of larger ethnographic collections or manuscripts. Nevertheless, there has been a scholarly development towards understanding Islamic amulets and talismans in terms of the material used, provenance, interpretation, and display [2, 3]. Additionally, it is important to understand how the practice and knowledge of amulets and talismans moved from one Islamic‐Arab‐and cultural tradition to another (e.g., Islamic‐African) and how this transition affected their materiality. The Islamic world is steeped in a rich history of material culture, including an enduring quest for spiritual blessing and protection that has produced a long tradition of a wide range of exquisite artIfacts [4]. Centuries before the introduction of block printing in Europe, amulet and talisman techniques with Arabic inscriptions, decorative motifs, and religious narratives were already in use in the Islamic world, differing mainly in their purpose, form, and cultural context. Although they often overlap in practise [5] and are still in use today [6] (e.g., the use of *ḥijāb*, *ḥirz*, *ṭilasm*, *tamīma*, *kharaza*, and *ruqya*). Both amulets and talismans have their roots in Islamic, Arabic, and African indigenous traditions [7] that include Qur’anic verses, numbered and lettered squares [8, 9], and local beliefs and are endowed with protective or empowering powers. In Islamic culture, amulets may bear, for example, Qur’anic verses, prayers, names of *Allâh* and/or the Prophet Muhammad, and religious tales and are carried in a pocket, or hung on traveling animals, worn under armor to be safe in battles [10], or swallowed or soaked in water to drink or poured over a person in which the ink of the text has been dissolved, or the smoke produced by its burning is inhaled to heal from illness. The main purpose of amulets is to bring the attainment of good luck in terms of health, possessions, wealth, and social relationships [11] as well as passive protection from the evil eye, demons, and supernatural beings (jinn). They were also used in popular belief as an alternative medicine [12]. The talisman is associated with a specified knowledge and is usually placed (hidden or buried) in specific locations and often created for a particular user for a specific purpose beyond mere protection, e.g., to achieve certain intended influences. Its power therefore exists from the moment it is activated, but it expires once its function is fulfilled. Talismans may contain a mixture of elements such as esoteric sciences (*ilm al‐huruf or ilm al‐ashkāl*), numerology, astrological symbols, magical formulae, or squares [13] or cryptic inscriptions that are believed to harness mystical powers [14]. Amulets and talismans were passed from one language, religion, culture, period, or region to another and consisted of different materials [15, 16]. The material aspects of paper amulets and talismans from the Islamic Empire can be secondary to their function, but they may also determine the way in which the amulet is used. The technology of Islamic papermaking has not yet been comprehensively analyzed either historically or scientifically [17]. Islamic‐African paper manuscripts were mainly studied for their philological, religious, and cultural significance with limited material analysis. In addition, they were analyzed sporadically, but comprehensive research on Islamic papermaking technology is still lacking comparing to the significant number of collections in the international GLAMs. Islamic manuscripts also have a particular challenge for analysis because they are often difficult to authenticate or date due to the complexity of the materials and recipes [18]. Unlike European paper, knowledge of Islamic papermaking techniques and materials remains incomplete and requires in‐depth research, both to enhance understanding of Islamic material culture and to improve conservation. Arabic amulet and talisman paper manuscripts and materials still require in‐depth research to deepen the understanding of Islamic material culture and improve conservation in European GLAMs (galleries, libraries, archives, and museums). The lack of contemporary scholarly research on Islamic paper collections held in GLAMs is worrying, especially with regard to art history, provenance, and authentication which are of great importance for curatorial, conservational, and scholarly reasons [19]. However, the art of Islamic papermaking extended from the western borders of China to Islamic Spain, from the eighth century AD to the seventeenth century AD or even later [20]. Due to trade and the decline of the paper industry in the Middle East [21], “quasi‐Islamic paper” was produced from the sixteenth century onwards, i.e, a hybrid paper in which the substrate is European but the finishing such as coloring, starch sizing and polishing is Islamic. The craft of papermaking then spread rapidly throughout Central Asia, the Middle East, and Africa, introducing new materials for writing (iron gall and black ink) and coloring (inorganic pigments such as iron and copper oxides and dyes) [22]. It is likely that many libraries and archives cannot afford to take adequate conservation measures based on technical analyses, which means that the remaining Islamic archival and library collections are at significant risk unless the technology of Islamic papermaking is sufficiently historically reviewed and scientifically examined. Over the decades, numerous analytical techniques have been used to identify and authenticate inks, pigments, and dyes in historical paper manuscripts [23]. However, the choice of approach, whether destructive or non‐destructive sampling, with more attention paid to the manuscripts, often remains questionable [24]. The involvement of knowledgeable, expert scientists is essential in any investigation of manuscripts, but the contribution from conservators, curators, and other specialists is paramount for a truly holistic assessment of the objects and their materiality, meaning, and context. There is no doubt that non‐invasive and non‐destructive techniques such as spectroscopic analysis are ethically preferable [25]. While Raman offers a relatively straightforward route to identification, and can record a distinctive fingerprint of both carbon‐based materials and iron gall inks distinguishing ink and colo recipes [26], the fluorescence spectra are often broad and overlapping, and their lifetime and intensity can change with pH, oxidation, or the presence of metal ions, making specific molecular identification difficult, especially for Islamic paper (and comparing to parchment support), which has a complex and heterogeneous composition and sometimes contains mixed non‐cellulosic materials, present in traces, such as additives and surface finishing. In the past, techniques such as Raman microscopy, surface‐enhanced Raman spectroscopy (SERS), fiber‐optic reflectance spectroscopy (FORS), UV–VIS reflectance, emission spectroscopy, and fluorescence spectroscopy have often produced inconclusive results [27, 28–29] and rarely applied to manuscripts of Islamic‐African context. However, the choice of technique must always be weighted against the value of the information that can be obtained from the analysis with the minimally invasive techniques being favored. In addition, the presence of certain materials on a manuscript can be used to infer the date and/or authenticity of the object in terms of the geographical origin and approximate date of invention of many older synthetic materials. Various aspects of a document, including the style, provenance, references to significant people or events, handwriting, type of carrier, watermarks, pigments, dyes and inks used, or sometimes even damage, can help with dating at least within a limited date range [30].

The nine principles of green heritage science [31] embody scientific analytical practices related to the economization of sampling in consistent with the ethical sampling guidance of heritage materials [25] promoting minimal use of sampling, waste generation, and energy consumption as well as the use of instruments with high selectivity and sensitivity. This protocol could also be enhanced by prioritizing the preferential use of imaging and mapping techniques [32, 33], such as hyper‐spectral imaging (HSI) for understating the spatial distribution and material properties of inks, pigments, and watermarks on the surface of historical manuscripts [34, 35, 36–37] and minimize the use of spatial elemental and molecular analysis techniques. HSI is a non‐invasive tool that advances the capabilities of near‐infrared (NIR) spectroscopy by simultaneously recording spectral and spatial information. It can be applied to materials such as inks, epigraphs, coloring materials, and paper manuscripts, mapping the distribution of different materials on a surface, thereby highlighting most alterations [38, 39]. When combined with machine learning methods, NIR spectral data can be successfully used for dating purposes, achieving unprecedented accuracy of up to 2 years using a well‐calibrated dataset [40]. In turn, Raman spectroscopy offers a relatively straightforward, non‐destructive route to identification and can record a distinctive fingerprint of both carbon‐based materials and iron gall inks (IGI). Moreover, researchers have recently hypothesized that it is possible to date manuscripts by analyzing the inks using Raman spectroscopy [41]. With the limitation of the non‐destructive analytical techniques, the use of destructive analysis such as fiber furnish, chemical spot tests, and pH can provide valuable information on the manufacture of manuscripts and assist in establishing its provenance [42]. Also, laser‐based analysis techniques such as laser‐induced breakdown spectroscopy (LIBS), laser‐induced fluorescence (LIF), and Raman spectroscopy, although microdestructive, are powerful tools for the scientific study of historical manuscripts, providing insights into their composition, origin, and preservation and contributing to a deeper understanding of these valuable cultural artifacts [43]. The application of these techniques will allow the characterization of the elemental and molecular composition of different types of materials such as inks, pigments, sizing, and the identification of stratigraphies of the polychromatic layers on the paper support, as well as the evaluation of degradation products [23, 43]. LIBS is a microinvasive technique that allows qualitative, semiquantitative, and quantitative material composition analysis. The analysis is done by the spectral analysis of the luminous plume generated by pulsed laser ablation of a small amount of material from the sample surface. Complemented with LIF in an entirely non‐invasive approach, analytical information on trace elements can also be obtained. However, few studies have employed LIBS, LIF, and Raman spectroscopies for the detection of trace elements in paper and ink samples. The combined application of the latter laser‐based techniques can show detectable and significant differences between modern and historical paper, inks, and pigments in terms of materiality, technology, and originality [44]. LIBS, LIF, and Raman analysis of historical manuscripts offer several advantages as it is micro/non‐destructive, i.e., it does not cause permanent damage to the manuscript and its materials. It provides fast results and can be performed in situ, reducing the need for extensive sample preparation. Additionally, LIBS enables the analysis of small areas or even individual particles, allowing localized investigations. By using these techniques combined to analyze historical manuscripts, researchers can gain insights into the materials and techniques used by scribes, illuminators, and illustrators in different time periods and geographical contexts. It can help to identify the presence of certain pigments, assess the stability of ink, recognize possible forgeries or alterations, and contribute to the understanding of historical writing and artistic practises. In this research, we aim to provide a comprehensive examination of a new type of Islamic‐African amulet and talisman paper collection in the Slovene Ethnographic Museum in Slovenia (SEM), which originated in the nineteenth to twentieth century in order to expand the knowledge about the materiality, authenticity, and symbolic interpretation of the collection. This study fills a critical gap by providing, to the best of our knowledge, the first scientific analysis conducted for amuletic and talismanic Islamic‐African paper collections, while this collection is considered to be the only Islamic paper manuscripts in Slovenia, providing an interdisciplinary impact by linking material analysis with historical, anthropological, and religious interpretation is of a great value to better understand the cultural significance of Islamic‐African talismans. The paper provides a better understand on how Islamic‐African amulets and talismans traditions reflect the interplay between Qur’anic orthodoxy and indigenous spiritual practices and what are their material characteristics using scientific and cultural analysis. The analysis focuses on the paper substrates and the inks used, highlighting recipes and symbolic use in Islamic/West African context which include both black and colored inks employed for writing and decorative purposes. The combined application of HSI, Raman, FTIR, LIBS, LIF, and fiber analysis overcome the known limitations of a single technique offering richer data with minimized sampling while providing information for preventive conservation of acidic and fragile Islamic papers in European GLAM institution.

## 2 Materials and Methods

### 2.1 Objects and Samples

The Slovene Ethnographic Museum (SEM) in Ljubljana, Slovenia, holds a unique historical collection of Islamic paper amulets and talismans. The collection was brought to Slovenia from Togo in summer 1914 by Anton Codelli from Ljubljana. In 1911, the German telecommunication company Telefunken entrusted to Anton Codelli one of the most demanding projects in the history of telegraphy for the establishment of a wireless connection between Berlin and the German colonies in Africa. The construction site was Kamina near the town of Atakpame, nearly 160 km from Lomé, the capital of Togo. The Codelli's colleague Leo Poljanec arrived from Ljubljana to Togo in 1912 and took over all commercial aspects of the construction site. They both traveled around the country, especially to northern Togo, taking photographs and collecting objects. The Codelli‐Poljanec collection of the SEM includes different ethnographic objects, around 600 photographs, and archival material. Among this collection are 80 sheets of amuletic and talismanic manuscripts, all bearing inscriptions. It is usually not possible to discern whether these objects were produced in Togo or were taken to there from other countries. To the best of our knowledge, this is the only known Islamic paper manuscripts in Slovenia. These artifacts are made of inked and colored paper in different sizes. They are characterized by Arabic text, inscriptions, numbers, and various geometric characters. For the purpose of this research, twelve objects (see Table 1) have been selected for detailed analysis based on its rich illuminations, themes, and purposes (the non‐selected sheets are so far similar to objects SEM EM 23 134–10 and SEM EM 23 134–25). In addition, historical samples from the Reference Historical Collection (fifteenth to nineteenth century from Central Asia and North Africa) [45] of the Heritage Science Lab of the University of Ljubljana (Slovenia) were used to support the interpretation of the spectroscopic analysis of SEM collections.

**TABLE 1:** Description of the selected objects from the SEM collection with their dimensions (L: length; W: width, T: thickness and RGB values (R, G, B)).

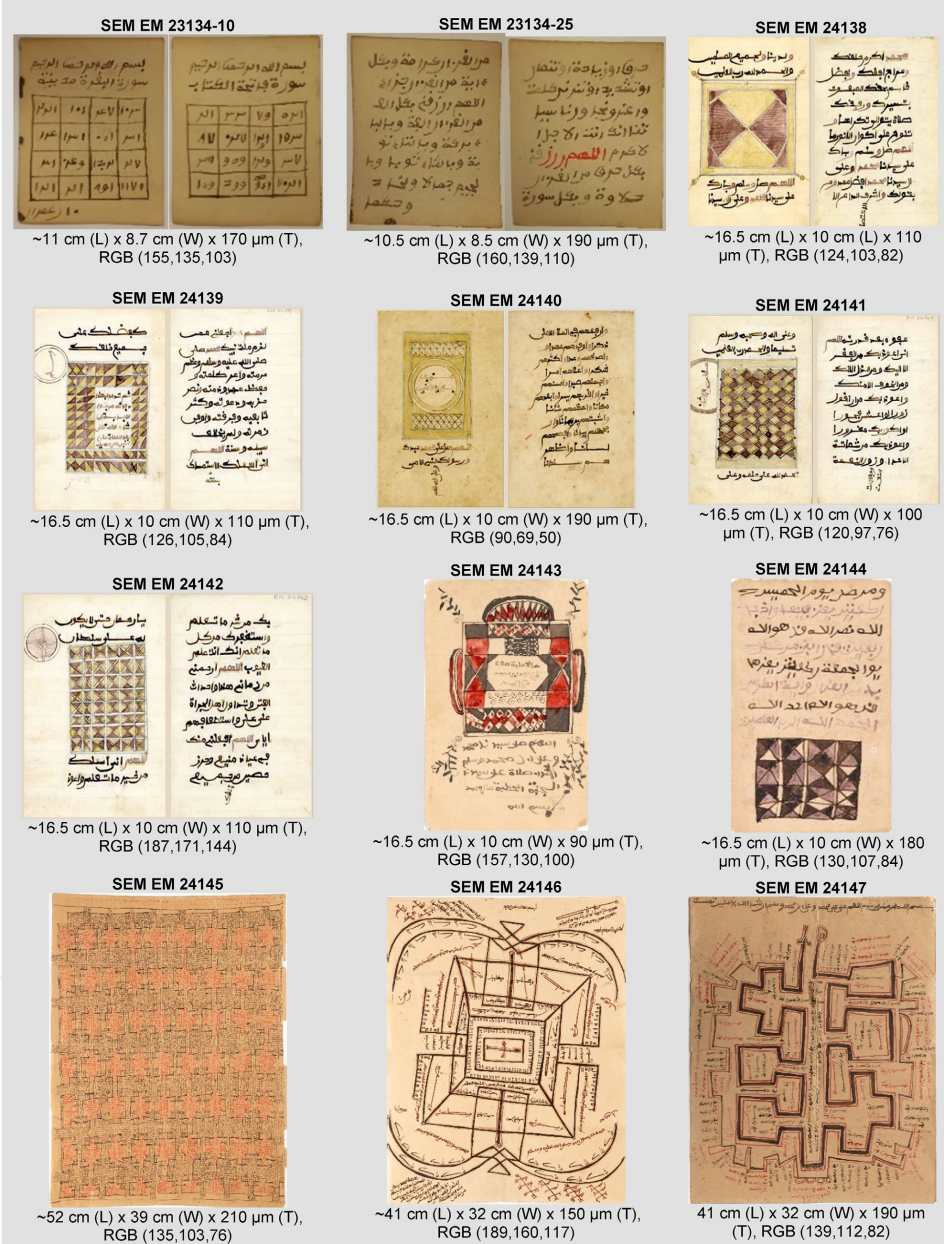		

### 2.2 Imaging and Microscopy

The Hirox HRX‐01 (HR‐2500E) digital microscope (Japan) with a 20x‐ to 2500x zoom range and various lighting options (dark field, bright field, polarized light, UV), along with a light board with cool LED transmitted light, and DSLR camera (Nikon 850D) have been used to document and capture the presence of watermarks and mould structure, e.g., chain lines in the paper support. The thickness of the paper support was measured using the digital micrometer Mitutoyo 500‐196‐30 ABSOLUTE AOS (±0.01 mm, US). The recently installed pushbroom Hyperspectral Imaging (HSI) scanner (ClydeHSI, UK) at the Slovenian node of the European Research Infrastructure (E‐RIHS.si) was used to collect spectral hypercubes in the 400–2500 nm range, using a translational stage and two cameras: VNIR (400–1000 nm) and SWIR (1000–2500 nm). Each camera features a 2D detector array, which simultaneously acquires one spatial dimension (*x*‐direction: 480/384 pixels) and one spectral dimension (260/288 wavelength channels) along the direction of the scanning stage (*y*‐direction). The current setup allows the scanning stage to accommodate objects up to a maximum size of A3 (~30 × 40 cm). Halogen lamps were used for illumination, warmed up for approximately 1 h before data acquisition to ensure stability. Whatman filter paper no. 1 was used as the background for all measurements. All spectral data were acquired in RAW format and spectrally and spatially corrected using a Spectralon reference standard. Proprietary software (SpectraSENS) controlled the entire acquisition and calibration process. Multivariate data analysis techniques, including principal component analysis (PCA) [46], were applied to the HSI data cubes to assist in the analysis and identify chemical similarities and variations among the objects. Table 10 in supplementary materials details the acquisition parameters used during the measurements.

### 2.3 Raman and Fourier Transform Infrared Spectroscopy

Raman spectroscopy analysis has been helpful in obtaining molecular information on inks composition. Punctual analysis was performed using a Micro‐Raman InVia Renishaw spectrometer equipped with a CCD camera electrically cooled. Raman analyses were conducted with a 785 nm diode laser and a diffraction grating of 1200l/mm. Laser power was up to 30 mW in order to avoid sample degradation. Exposure time and accumulations were optimized depending on each material. Raman examinations were performed on the samples without any previous preparations. A Bruker Alpha II FTIR spectrometer, equipped with an external PC, was used in attenuated total reflectance (ATR) mode to analyze samples in the mid‐infrared region, ranging from approximately 4000 to 400 cm^−1^. Each sampled spot was averaged over 3–5 measurements, followed by baseline correction and normalization using the OPUS software.

### 2.4 Laser‐Induced Fluorescence (LIF) and Laser‐Induced Breakdown Spectroscopy (LIBS)

Laser‐nduced fluorescence (LIF) spectra were collected using laser excitation at 266 nm (4th harmonic of a Q‐switched Nd:YAG laser, 6 ns pulses, 2 Hz repetition rate) and a 0.30 m spectrograph with a 300 l/mm grating blazed at 500 nm (TMc300 Bentham) coupled to an intensified charged coupled detector (ICCD, 2151 Andor Technologies) providing a spectral coverage of 280 nm at each laser pulse. The LIF spectra were performed on paper‐based samples with different inks. The temporal gate was operated at zero‐time delay with respect to the arrival of the laser pulse to the surface of the sample and with a width of 3 μs. The samples were illuminated by the laser at an incidence angle of 45° with pulses of around 0.1 mJ. For the results presented here, 300 and 360 nm cut‐off filters were set in front of the spectrograph entrance to avoid the scattered laser light from the surface of the samples and to avoid second‐order diffraction. Each spectrum resulted from the accumulation of 15 measurements, in the range of 300–750 nm, using a spectral resolution of 5 nm. LIBS analyses were carried out using the same laser source as for LIF measurements. The spectra were recorded using a 0.2 m Czerny‐Turner spectrograph (Andor, Shamrock Kymera‐193i‐A) equipped with a grating of 1800 grooves/mm (blazed at 265 nm) and coupled to a time‐gated intensified charge‐coupled device (ICCD) camera (Andor Technology, iStar CCD 334, 1024x1024 active pixels, 13 × 13 μm pixel). The laser beam was directed to the surface of the paper‐based samples using mirrors at an incidence angle of 45°. LIB spectra were recorded in the 240–600 nm wavelength range using a step‐and‐glow mode at intervals of 30 nm. Each LIB spectrum corresponds to individual acquisition at a 0.17 nm resolution with a gate delay and width of 200 ns and 3 μs, respectively. A cut‐off filter at 300 nm was placed in front of the entrance window of the spectrograph to reduce the scattered laser light and to avoid second‐order diffractions.

### 2.5 Paper Fiber Analysis

To gain an overview of the condition of the collection and to economize the object sampling, six representative samples were selected for destructive analysis using different analytical methods. The aim was to check object fragility through its acidity and identify fibers and sizing used. The pH was determined using the cold extraction method, where each sample was immersed in deionized water and left overnight in a sealed container. The measurements were performed at 25°C using a TOLEDO SevenCompact S220 pH Benchtop Meter paired with an InLab_Micro pH combination electrode, which was calibrated with four buffer solutions (pH 2, pH 4, pH 7, and pH 10). All values were rounded to the nearest 0.1 pH unit where samples (1 mg of paper fibers in 0.1 ml distilled water) were taken out of the extraction liquid during measurement. For identification of the fibers sources (e.g., wood and linen), the six samples used in pH analysis were washed by distilled water, dried in room temperature, and used for fibers furnish analysis. The standard procedure was followed using the ISO 9184‐1:2023 [47] to investigate fiber composition. The samples were examined using microscope Nikon Eclipse 80i equipped with a DS Camera Head DS‐5 M under transmitted light with staining process, using reagent Graff C. Fiber identification was based on morphological and color shade characteristics. For counting and identification of fibers, magnification of ×100 to ×200 was applied. To calculate the fiber content in the sample, we used the following weighting factors: coniferous (unbleached): factor 1.0; straw: factor 0.4; mechanical wood pulp: factor 1.3. Starch is one of the characteristic materials in Islamic papermaking as a sizing material. The iodine test [48] was used to establish the presence of starch. The I_2_ solution was prepared by adding 5 g of I_2_ (≥99.8%, Sigma Aldrich, Dorset) to 10 g of KI (crystals, Sigma Aldrich, Dorset) dissolved in 100 ml of deionized water. After dissolution, the prepared solution was diluted 1:4 before use. One drop of the diluted solution was then added to less than 0.01 mm^2^ of paper sample. Color change was observed under low magnification.

## 3 Results and Discussion

### 3.1 Interpretation Context

#### 3.1.1 Object SEM EM 23 134‐10

This talismanic object is an example of another similar 57 sheets with magic squares on both sides that are handwritten in a good quality, probably in Moroccan calligraphy. The text is written in black ink on both the recto and verso following the same pattern: first line: *bismillah al‐rahmân al‐rahîm* (In the name of *Allâh* [the only God] the Beneficent, the Merciful) and second line: title of the chapter in the Qur’an usually followed by the name of the place where it was revealed, either Mecca or Medina [*Sūrat Fātiḥa Al‐Ketab and Sūrat Al‐Baqar*a (Madinah/Medina), with the addition of a magic square with a specific combination of letters and numbers [49]. The title of the next chapter is written below the square at the end of the verso (like a pagination). The most popular in the Islamic world is the 16‐magic square (four‐by‐four vertically and horizontally) usually containing chessboard‐like compartments in similar size, each containing a number and/or letters. According to the Abjad numerals (*Hisab al‐Jummal*) [13], each Arabic letter has a numerical equivalent and vice versa and their alphanumeric results remain consistent across the horizontal, vertical, and diagonal lines with a mathematical equilibrium representing *a’dad al‐wafq* ‘harmonious dispositions of numbers’. Magic squares often also contain textual clues, such as phrases and sounds that should be spoken aloud for the protective power to take effect.

#### 3.1.2 Object SEM EM 23 134‐25

This amuletic object is a sample of 11 similar handwritten sheets (with only Arabic text on both sides, of almost the same size, as object SEM EM 23 134‐10, written in a good quality (probably Moroccan calligraphy). The text is written in black ink (only the keyword *Allâh* is rubricated in red [50] and is inadvertently continued halfway through the next word); red inks have been frequently used in Islamic manuscripts to mark important titles, rubrication, or sacred names. The text is written in 7 lines on both the recto and verso following the same pattern and contains prayers and supplications (asking for blessing while reading the Qur’an) that do not contain consonantal diacritics or symbols to separate the verses. It appears that these sheets were part of a notebook, as a paper book binding was found in the collection that was not sewn, which is typical of African amulet books.

#### 3.1.3 Objects SEM EM 24 138 to 24 142

This group of objects consist of five sheets (10 pages) of similar dimension and handwriting as well as ink (black, red, brown, and yellow). Both the recto and verso contain handwritten text by skilled calligrapher (probably Moroccan calligraphy) while one page is decorated also with a square as a center point colored in brown and yellow. The objects represent five amuletic sheets of an illustrated manuscript copy from the 19^th^ of the book ‘*’Dalâ’il al‐khayrât* (*Waymarks of Benefits*), which originally written in the 15th C. by al‐Jazuli (c 870/1465), and well known in the Islamic Sufism, containing prayers and supplications (probably for each day of the week) in honor of the Prophet Muhammad. This book consists of unbound (loose) sheets and is often worn by a one person in West Africa, which is typical to western Africa [11]. The text contains prayers and supplications in honor of the Prophet Muhammad in which the name of *Allâh*, Muhammad, and the *Hamza* over the letter Alif (ألف) and the letter (و) are written in a different color (brownish ink) along with other letters by mistake of the calligrapher. There are no consonantal diacritical marks, and no symbols to separate the verses. In the verso of some of the objects, there is also spherical shape next or in the square contains some words (e.g., the name of the day such as Thursday). However, the handwritten texts were probably written by the same calligrapher. According to Hames [11], a typical feature of Qur’ans from Western Africa is that they are not bound but consist of loose sheets. This style of decoration and colors are typical of Qur’ans from West Africa in the region between Mali, Niger, and Chad, which are based on originals from the Almohad dynasty (twelfth to thirteenth century).

#### 3.1.4 Object SEM EM 24 143

The object is decorated sheet on one side only of prayer with a text in black ink and multicolored motives and vegetable floral designs (black and red). The handwritten text was executed in a poor quality. At the bottom, there is *tasliya* [51] (supererogatory prayer) and *basmala* (in the name of *Allâh*, the Beneficent, the Merciful) prayers and supplications in honor of the Prophet Muhammad.

#### 3.1.5 Object SEM EM 24 144

The object is a decorated sheet with handwritten prayers in black and purple inks on only one side. It may contain litanies/prayers for each day of the week (e.g., Thursday and Friday). The text is executed in the top of the page by skilled calligrapher (probably Moroccan calligraphy), while the lower part is decorated by a colorful square as a center point colored in black and purple inks. There are no consonantal diacritics, and there are also no symbols to separate the verses.

#### 3.1.6 Object SEM EM 24 145

Inscribed on one side by a skilled calligrapher, the object is a large talismanic chart with colorful textual and graphic repetitive motifs of joined and crossed rectangles, the main purpose of which is to attract *rizq* (sustenance), *baraka* (blessing), and success in commerce and trade [11]. In many West African Islamic traditions, particularly among Sufi communities, trade and wealth are not considered as worldly vices, but blessings from *Allâh* if they are ethically persued. The red inscription at the beginning says: “To whoever will wear this talisman […], *Allâh* will open the doors to wealth and trade. The inscriptions *Allâh*, *yâ Allâh*, *yâ Allâhumma*, and the Sublime (*‘al‐azîm*) are repeated in brown and red. At the top, there is *basmala* (In the name of *Allâh*, the Beneficent, the Merciful), *tasliya* [51] (supererogatory prayer), and a supplication for *Allâh* to open the doors of giving during trade. The use of the name (*‘al‐azîm*) reinforces the power of the talisman, while the invocative forms of the divine names, which invoke the presence and power of God aligning with the themes of abundance, security, and divine favor. The graphic motifs of linked and crossed rectangles are probably symbolic in Islamic‐African traditions, particularly in Mali, Niger, and Sudan and Nigeria which support the object origin [2]. These talismanic grids (*wafq or khatam*) are often used in numerological magic squares, Qur’anic verses, or coded letters. The use of crossed rectangles can symbolize protection and enclosure (a sacred space around one's possessions or property) or barriers against envy (*hasad*), theft, or spiritual harm. In addition, it could also symbolize flow or connectivity possibly representing trade routes, networks, or cosmic connections.

#### 3.1.7 Object SEM EM 24 146

Written on one side by skilled calligrapher, the talisman is an example of adaptive spirituality that uses quartic authority, angelic mediation, and local beliefs to create multilayered defense mechanism. Its design reflects both universal Islamic principles and the cultural characteristics of the West Africa region. The object is a large talisman chart of colorful textual and graphic designs in black and red. In the center is a cross‐shaped inscription of *Muhammad* surrounded by a symmetrical graphic. The centering of Prophet Muhammed's name emphasizes his role as an intercessor and source of blessing and anchor the spiritual authority of the talisman. The sheet contains the name of God, the Prophet, angels, citations and symbols from the Qur’an, and local magical entities typically used in West Africa and the Maghreb, as a protective fort against lies and defamation [11]. The mention of archangels such as Jibril, Mikail, and Israfil is common in Islamic esotericism for protection and symbolizes celestial guardship. There are ‘verses’ (*ayât*) from the Qur’an: suras (*sűra* 112, *al‐Ikhlâs*), *basmala;*, the names of archangels, names of God [(*Wahhâb*, the All‐Giving), *Kâfî* (the Sufficient), and *Shâfî* (the Healer)], names of prophets, e.g., Moses), repetition of two Arabic letters K (*kâf*) and Sh (*shîn*), and names of local jinns [2]. The use of the Prophet Musa's name could be due to his story of overcoming adversity. The repetition of the letters K (*kâf*) and Sh (*shîn*) could be related to the Arabic numerology, where each letter has a value and esoteric meaning. Alternatively, the two letters could be abbreviations or parts of other words or names, e.g., *kâf* for *the Kâfî* (the Sufficient one) and *shîn* for the *Shâfî* already mentioned here in the text here*.* The repetition of these words could amplify their power. The use of local jinns represents folk practices in West Africa, where it is believed that jinns can be controlled or petitioned by talismans. The inclusion of jinns suggests a parametric mix of Islamic and pre‐Islamic practices, with Islam discouraging engagement with jinns invoking their protection or neutralizing their harm.

#### 3.1.8 Object SEM EM 24 147

The object is a large talismanic chart written on one side by skilled calligrapher with colorful textual and depicted graphic design using the name of the Prophet Muhammad as a seal in the form of a fort or a sord turned in all directions for protection, probably implying all‐around protection that keeps the wearer safe from unseen harm, envy, or spiritual attack. The fort stands for spiritual defense and protection, with the protection of God surrounding the believer. The sword in Islamic iconography can symbolize strength, authority, and victory over evil or misfortune. There is an inscription of the word ‘*yâ Allâhi’* (oh, *Allâh*) in red and black. The drawing begins with the first letter (M/*mîm*) and ends with the last letter (D/*dal*). At the top are *basmala* (In the name of *Allâh*, the Beneficent, the Merciful), *tasliya* (supererogatory prayer), *inshāʾa Allāh* (*Allâh* [God] willing), *amîn* (amen), a liturgical seal of the prayer that affirms belief in the efficacy of the supplication, and *tammat* (it is accomplished), which signifies the ritual closure, completeness, and integrity of the talisman. The use of the red color often serves to invoke power, urgency, or divine energy to intensify the supplication. The repetition of this black and red invocation is meant perhaps to reinforce the constant pleasure for divine help and connection.

### 3.2 Paper Support

#### 3.2.1 Morphology

**FIGURE 1:**
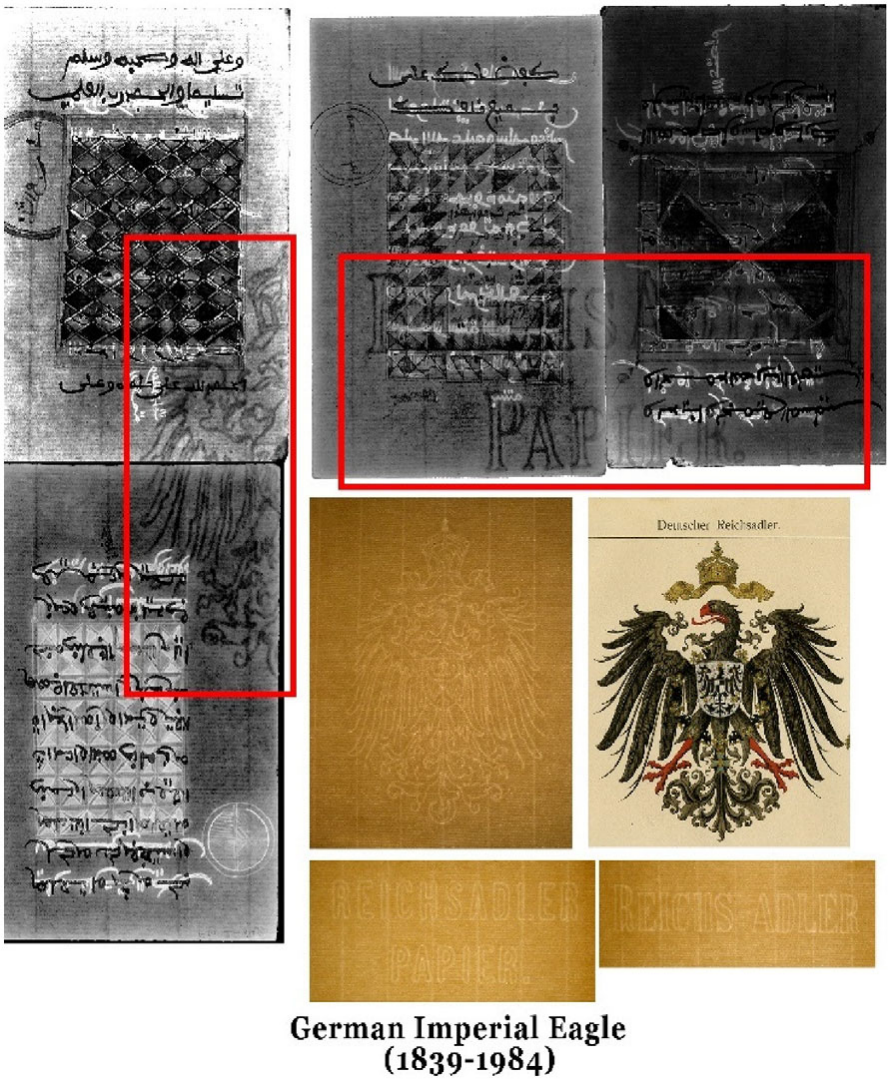
Watermark and countermark in the objects—SEM EM 24 138 to 24 142 (excluding 24 140)—of a German mill (German Imperial Eagle) where a single sheet is cut into four prices.

The photographic and microscopic investigations of the objects provided information about their morphological features, e.g., mould structure, watermarks, thickness, and dimensions. Regarding physical features, the objects can be grouped into four size categories (width × length): approximately 8.5 × 11 cm, 10 × 16.5 cm, 32 × 41 cm and 39 × 52 cm. Their thickness varies, ranging from 90 to 210 µm. Two of the objects (SEM EM 24 145 and 24 146) are made of wove paper while the rest are laid paper, except for SEM EM 24 143 which shows a wire‐mesh pattern indicating machine‐made origin. Most of the laid paper objects share similar mould characteristics, typically showing four laid lines spaced ~25–27 mm apart along with chain lines 7–8 per 1 cm separated by ~1–1.2 mm. An exception is SEM EM 24 147, which, due to its larger length, shows 15 laid lines with same spacing of approximately 26–27 mm. The visual appearance of the objects reflects differences in manufacturing and finishing processes, including fiber composition and surface treatment, e.g., sizing. Some objects, such as SEM EM 24 138, 24 139, 24 141, 24 142, and 24 146, appear creamy in color with a smooth surface. In contrast, others are darker and visually exhibit signs of mechanical degradation, particularly SEM EM 24 145. Watermarks were identified in only seven objects where SEM EM 23 134–25 and SEM EM 24 147 share the same unrecognized watermark, possibly of British origin. A group of objects—SEM EM 24 138 to 24 142, excluding 24 140—share the same watermark and countermark of a German mill featuring the German Imperial Eagle (used between 1839 and 1984) (see Figure 1). Typical to African manuscripts [52], the watermark found in groups assuming that a single hand‐made sheet was cut into four or more pieces. Although object SEM EM 23 134–10 does not show a watermark, some of the other similar sheets of the same group (57 sheets in total) have the same watermark of the British origin. The same watermark found in object SEM EM 23 134–25 was also identified in 11 other sheets from the same group in the SEM EM collection.

#### 3.2.2 Fibers and Acidity

In view of the variations in color and physical conditions of the paper sheets and the fact that some objects showed distinct characteristics from the others, pH measurements and fiber furnish analysis were carried out on selected objects with minimal sampling. The aim was to gain an insight into the types of paper used in the countries of origin in order to provide evidence on the provenance of the objects [53]. Only six samples were selected (SEM EM 24 140, 43, 44, 45, 46, and 47) where both analytical techniques were applied to the same samples to avoid sample waste, starting with pH measurement followed by fiber identification. As expected, the samples proved to be quite acidic, with pH values ranging from 4.8 to 5.3. Due to the limited sample size, the paper samples for the fiber furnish analysis were crushed using the surface‐scraping method instead of the standard method of disintegration with a disintegrator then samples were stained and observed under a microscope. The paper samples were quite compact, with mostly shredded fibers on a short dimension. Most samples had similar fiber composition, particularly SEM EM 24 140, 43, 44, and 47 which were identified as a mixture of wood pulp (~60–70% softwood, conifer fibers) and straw fibers perhaps from the paper mill waste of (~30–40%). SEM EM 24 146 had a similar fiber composition but with a higher proportion of softwood and less straw and appeared to be in better condition (pH 5.3) compared to the other large sheets in the collection. In contrast, SEM EM 24 145, the most brittle sample (pH 4.9), had a mixed furnish of softwood (47%) and hardwood (53%) as well as grass fibers (likely straw or leaf deciduous fibers). Hardwood or deciduous trees have shorter fibers that give a smoother texture and a better writing surface, and they can be sourced locally or mixed with imported pulp [54]. However, the use of coarser and more brittle grass/straw fibers, which are in low abundance in Africa and the Middle East, or the reuse of lower quality pulp could lead to brittleness of the object. Overall, the fiber analysis, acidity and physical appearance (brittleness) in some samples suggest that the paper substrates were of relatively low quality. While these findings are consistent with the use of inferior paper materials, particularly in samples with higher proportions of straw and hardwood fibers, further investigation with a larger sample set would be necessary to confirm this interpretation.

#### 3.2.3 Sizing and Additives

**FIGURE 2:**
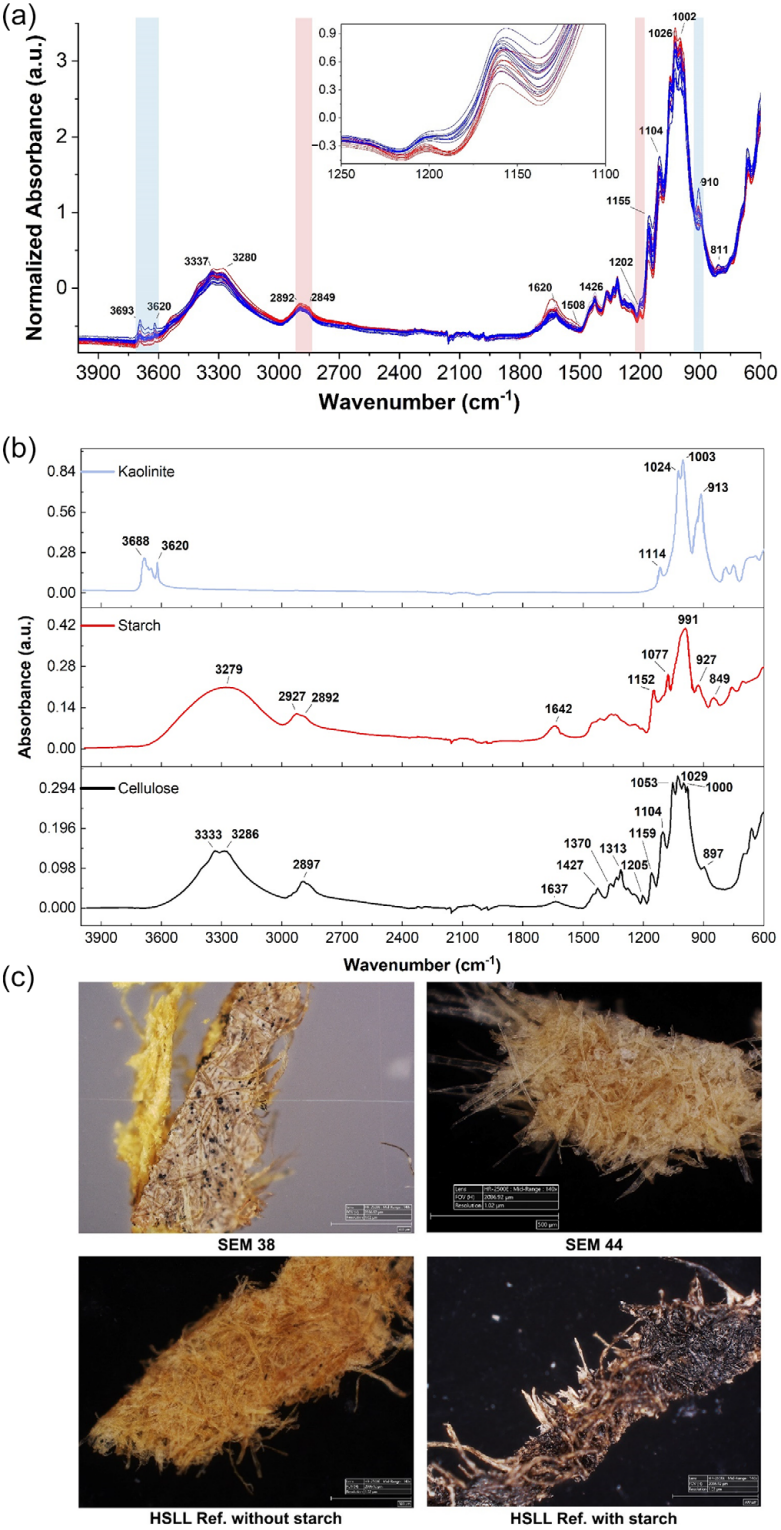
(*Top‐a*) FTIR spectra (normalized by SNV) of 12 objects (both sides) in the range of 4000–600 cm^−1^, categorized by color according to starch presence determined by the iodine test (red: with starch; blue: without starch). Characteristic bands for a potential filler (Kaolinite) are highlighted in light blue. Areas highlighted in light red indicate observed differences between samples with and without starch. (*Middle‐b*) FTIR spectra of reference materials (Kaolinite, Starch, and Cellulose) from the online database of the University of Tartu [56], showing their characteristic bands. (*Bottom‐c*) Microscopic examination of four samples: SEM 38 showing two faces (one with starch, one without), and SEM 44 showing traces of a coating or filler observed in several samples; two historic HSLL samples (AP91 and AP65) after iodine staining, indicating the presence or absence of starch.

**FIGURE 3:**
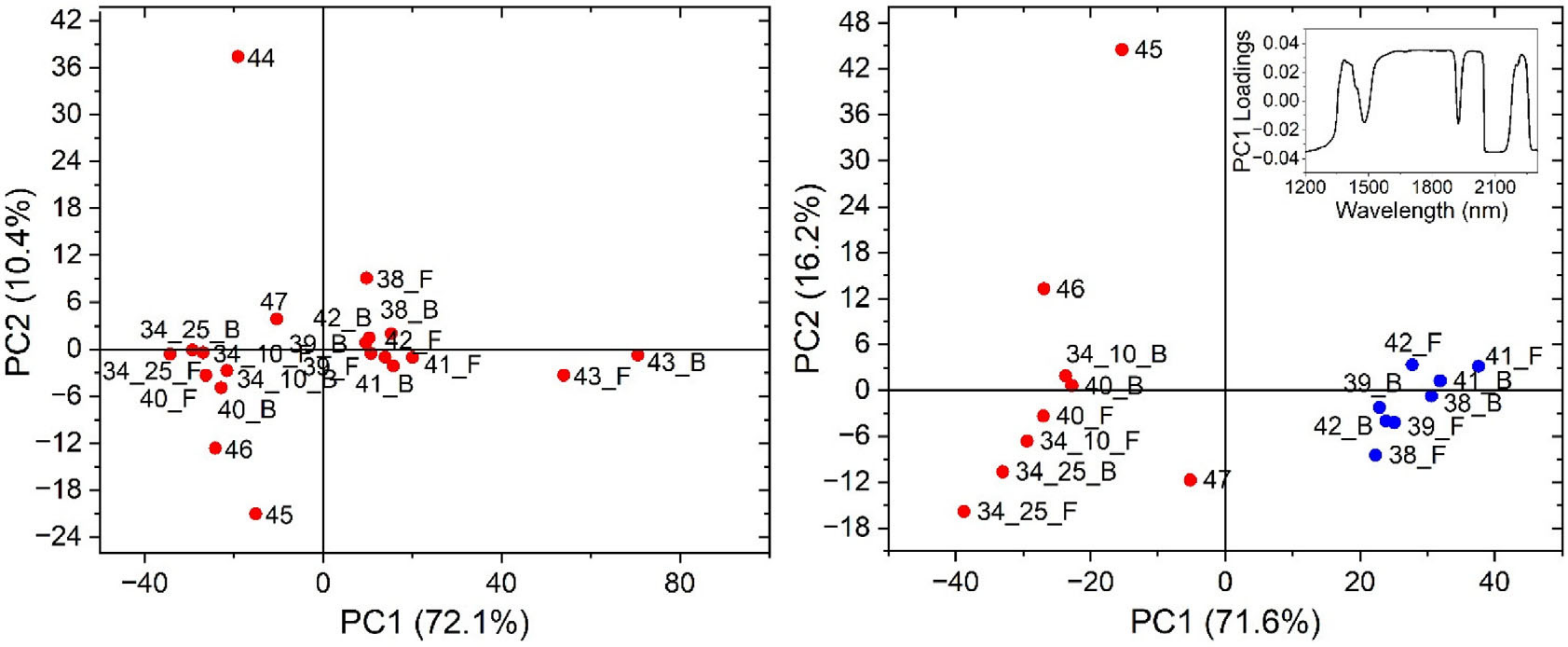
PCA score plot (PC1 vs. PC2) based on the NIR spectra extracted from the HSI SWIR data cubes (1000–2500 nm) of all the objects (left) and after the elimination of Object EM 24 143 and 24 144 (right). F and B represent the front and back sides of the object.

Starch sizing, as one of the characteristics of Islamic paper [48], was detected using the iodine test in six objects SEM EM 23 134‐10, 23 134‐25, 24 140, 24 144, 24 147 and on one side of SEM EM 24 138 showing various reactions with the iodine solution (perhaps due to the source and quantity of starch used) [55]. However, the starch sizing can be added to the European paper sheet by a local craftsman [17]. The findings were further investigated by the FTIR‐ATR analysis (Figure 2. top) of the objects which showed the presence of starch by virtue of bands, although its spectral features were partially obscured by the overlap with cellulose bands. Some spectral regions have showed separation between samples with and without starch particularly in the range 1000–1200 and 2850–2900 cm^−1^ after SNV normalization as shown in Figure 2. Additional coatings or fillers were also identified, such as kaolinite (at ~912 and 3620–3690 cm^−1^) which was consistently observed across all objects and confirmed by microscopic examination (Figure 2. bottom). A peak around 1510 cm^−1^ was more clearly observed in some samples (SEM EM 24 143, 24 144, 24 145), indicating the presence of lignin and confirming the use of wood pulp. No distinct proteinaceous peaks were detected. Although resins such as rosin in combination with alum may have been used as sizing agents, their presence could not be confirmed from the FTIR‐ATR data alone. LIF measurements from substrate paper samples (SEM EM 23 134‐10 and 25, and SEM EM 24 140, 41, 43, 44, 46 and 47) upon laser excitation at 266 nm (Figure S1) have revealed similar spectral behavior (broad bands ranging from 300 to 750 nm) with differences in their intensities which could be due to the materials used in their manufacture or state of degradation [57]. On the other hand, the sample SEM EM 24 145 shows a different LIF spectrum, compared to the previous ones, with spectral broadening and red shifting, confirming the nature of its state (dark color and high degree of degradation) already indicated in subsection (fibers and acidity) that could be due to higher aging and oxidation effects. On the other hand, in the PCA score plot (PC1 vs. PC2) (Figure 3) based on the NIR spectra extracted from the HSI SWIR data cubes (1000–2500 nm) of the objects, objects SEM EM 24 143 and 44 clearly stood out from the rest, indicating distinct chemical properties. When these two were excluded, the remaining objects separated into two main groups along PC1. SEM EM 24 138, 39, 41, and 42 clustered together, which aligns with their morphological similarities. Another group included SEM EM 23 134‐10, 23 134‐25, 24 140, 47, and 46. Notably, SEM EM 24 145 appeared as an outlier, possibly due to differences in fiber composition, supported by the presence of a lignin peak in its ATR‐FTIR spectrum.

#### 3.2.4 Inks

Identification of inks and their distribution is crucial not only for understanding the provenance and cultural context of objects but also for informing appropriate conservation strategies. These inks include carbon‐based inks, iron gall inks, plant‐based dyes, and mineral pigments, which are often used alone or in mixtures. In order to gain insight into the variations of inks on the objects and to optimize sampling and localized measurements, HSI was first performed on all objects, allowing non‐invasive visualization and mapping of different inks and possible mixtures across the surfaces and guiding the selection of points for more detailed identification using analytical techniques such as ATR‐FTIR, Raman spectroscopy, LIBS, and LIF. Visually, all objects show the use of dark ink (black with varying hues), either alone—as in SEM EM 23 134‐10—or in combination with red ink, as in five objects SEM EM 23 134‐25, 24 143, 45, 46, and 47. In addition, six objects (SEM EM 24 138, 39, 40, 41, 42, and 44) show the presence of other inks or mixtures, including yellow, purple, and brown ink. The HSI data cubes in the 1000–2500 nm range revealed a spectral signature of the inks on the entire surface of all objects, with a sequence of grayscale images representing the reflectance of the material in each band. A false‐color image based on PCA is generated from the data cubes to visualize and identify the chemical variations between the inks. In general, black inks with iron gall ink and carbon‐based inks can be distinguished, which have distinct spectral behaviors. IGI tends to fade or disappear around 1700 nm, while carbon‐based inks remain dark over the entire spectral range [58].

#### 3.2.5 Dark Inks

**FIGURE 4:**
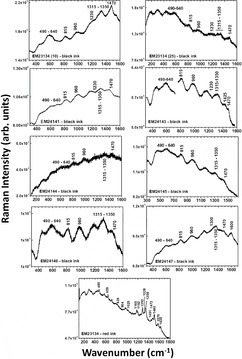
Raman spectra with characteristic bands of black iron gall ink (IGI) and red ink.

**FIGURE 5:**
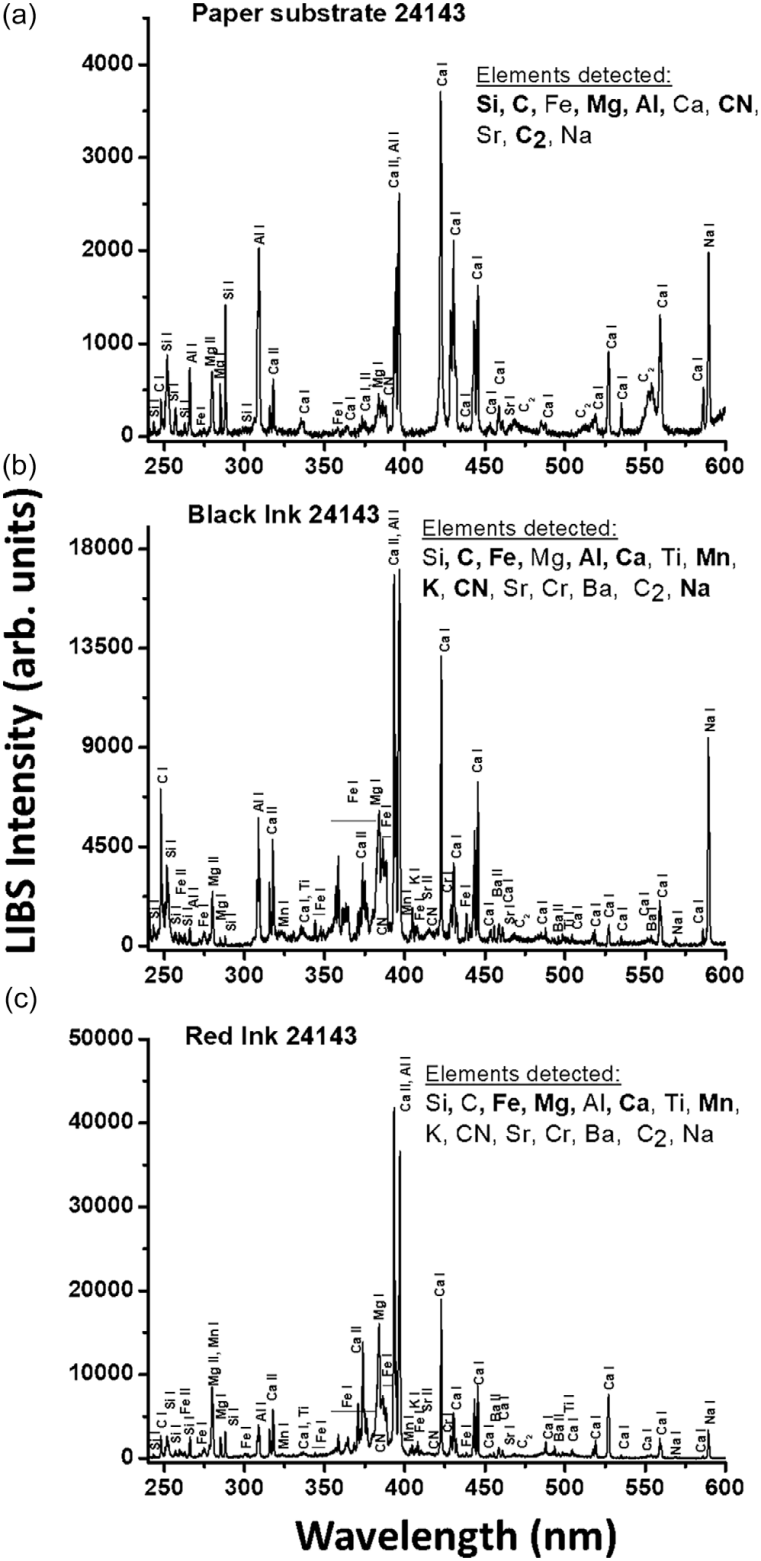
LIBS spectra from different ink spots in sample SEM EM 24 143. (a) Paper substrate, (b) black ink, and (c) red ink. It corresponds to a single laser pulse. The time delay and gate width were 200 ns and 3 μs, respectively. The assignment of the line emissions is done using the NIST database (https://physics.nist.gov/PhysRefData/ASD/lines_form.html). The main detected components are indicated in bold.

**FIGURE 6:**
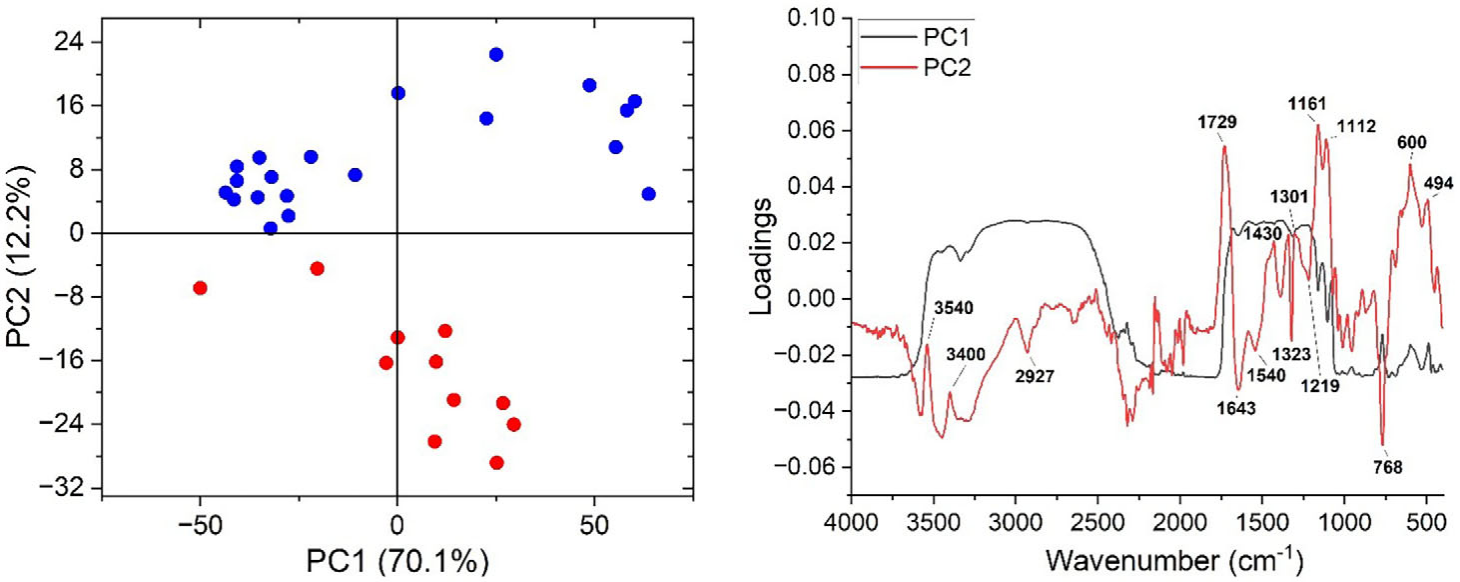
PCA of EM objects based on the NIR spectra of dark inks or black ink extracted from the HSI datacubes (left: PC1 vs PC2) and the loadings of the two PCs (right). The analysis revealed that samples can be divided into two groups along PC2, based on key peaks include 2927, 1730, 1640, 1540, 1430, 1320, 1112, 768, and 600 cm^−1^ which reflect differences in ink composition, degradation state, and possible additives.

**FIGURE 7:**
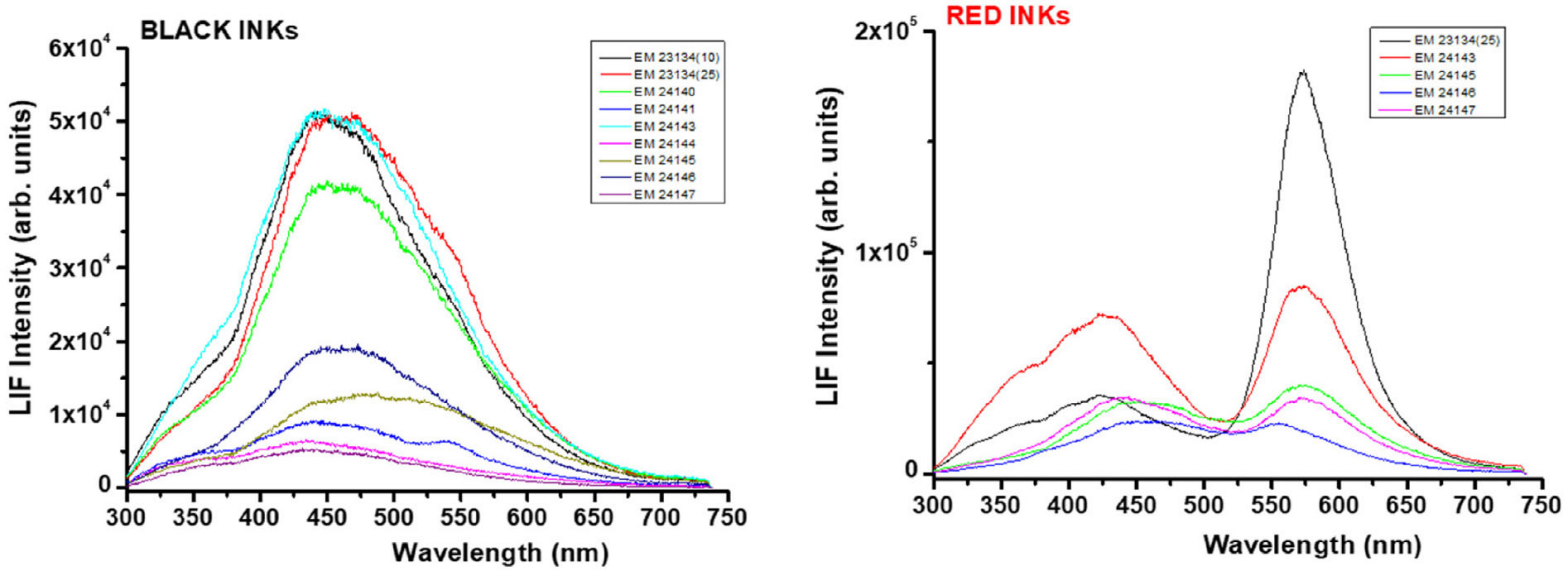
LIF spectra of black (left) and red inks (right) upon laser excitation at 266 nm. LIF spectra correspond to the accumulation of 15 measurements using gate delay and width of 0 and 3 µs, respectively.

The HSI images of the SEM EM objects indicate the use of iron gall ink for all dark writing and drawings. The borders in objects SEM EM 24 138–42 were probably drawn with carbon‐based or mixed ink. Object SEM EM 24 145 showed some additional ink in few locations, probably carbon while SEM EM 24 146 showed traces of iron gall ink mixed with red ink in some text. Ink smearing was observed at the edges of some objects likely due to calligraphy mistakes, while no missing or overwritten scripts or evidence of ink migration was captured [59]. The ink was also heavily applied at some letters which provided good thick ink spots for later spectroscopic analysis to minimize interference of the paper substrate [60]. Micro‐Raman measurements (Figure 4) confirm the use of iron gall ink in all tested objects identified by the characteristic bands observed at 1470 (strong), between 1350 and 1315 (strong), 1230, 960, 815, and 640–490 cm^−1^ (broad due to multiple peaks) characteristic of the metal polyphenol complex. In addition, the observation of the main three Raman bands at 1470, 1315–1350, and 490–640 cm^−1^ is a positive indication of the presence of iron‐gall ink [61, 62]. Moreover, carbon black ink is also observed in the object SEM EM 24 147 by the presence of typical broad bands centered at 1300 and 1600 cm^−1^ due to the amorphous carbon which are assigned to *ν*(C—C) and *ν*(C=C) vibrational modes, respectively. This may indicate the use of mixed inks for this object. Raman bands shift from 1478 to 1470 cm^−1^ observed for the analyzed objects can indicate possible degradation of the IGI due to the increasing pH [63]. Due to the micro‐destructiveness of LIBS analysis, it was used only on limited spots and smeared black inks at the edge of the paper sheets of objects SEM EM 23 134‐10, 23 134‐25, and 24 143. LIBS spectrum of the black ink from sample 24 143 shows (see Figure 5b) the presence of the elements of silicon (Si), carbon (C), magnesium (Mg), aluminum (Al), calcium (Ca), iron (Fe), manganese (Mn), potassium (K), strontium (Sr), titanium (Ti), chromium (Cr), barium (Ba), CN, C_2_, and Na, where C and Fe constitute the main elements which is similar to the elemental composition of reference model IGI (Si, C, Mg, Fe, Ca, Al, CN, Sr, Ba, C_2_, H, and Na). C, CN, and C_2_ represent the major organic compounds of the ink, e.g., tannins, Arabic gum, or substrate paper (Figure 5), while Fe is the main ingredient of IGI from ferrous sulfate and Mg is also a trace element often present with iron ores. In addition, Ca and Mg were present, presumably as in their carbonate form, as an additive in the paper pulp [64]. Al and K could be from alum which was likely added to ink as a mordant [63], and the rest of elements are part of possible impurities. FTIR analysis of black IGI shows characteristic features associated with its components. A broad O‐H stretching peaks at 3400 cm^−1^, from phenolic hydroxyl groups in the tannins. Strong, broad bands at 1000–1200 cm^−1^ attributed to C‐O, C–C, C–H, and C‐OH vibrations and strong absorption bands around 1311–1315 [65] along with Fe‐O peaks at 531, 555, and 662 which may be associated with Fe^3+^‐tannin complexes. Additional FTIR peaks at 1368 and 1424 may indicate ink degradation products, e.g., organic acids or calcium oxalate [66] (see Figure S2). To explore compositional variation, PCA was applied on FTIR spectra collected from different inked areas of the objects for the dark ink. The analysis revealed that samples can be divided into two groups along PC2, based on key peaks include 2927, 1730, 1640, 1540, 1430, 1320, 1112, 768, and 600 cm^−1^ which reflect differences in ink composition, degradation state, and possible additives (Figure 6). LIF analysis of black IGIs shows, in the region around 400–500 nm, a band can be assigned to inorganic compounds of the iron gall ink attributed to iron oxide or Fe^3+^/Fe^2+^ ions. LIF spectra from dark inks show broad spectral emissions in the range of 300–750 nm centered at 450 nm with different shoulders around 350 and 500 nm attributed to the chromophores Fe^3+^/Fe^2+^ [67] (Figure 7), Top graph)). The sample SEM EM 24 146 reports additional band around 550 nm revealing the presence of an extra colorant (red ink) in the composition of the dark ink as confirmed by HSI. The same behavior has been shown by the SEM EM 23 134‐25 object although in this case with slight contribution of the red ink.

#### 3.2.6 Colored Inks

**FIGURE 8:**
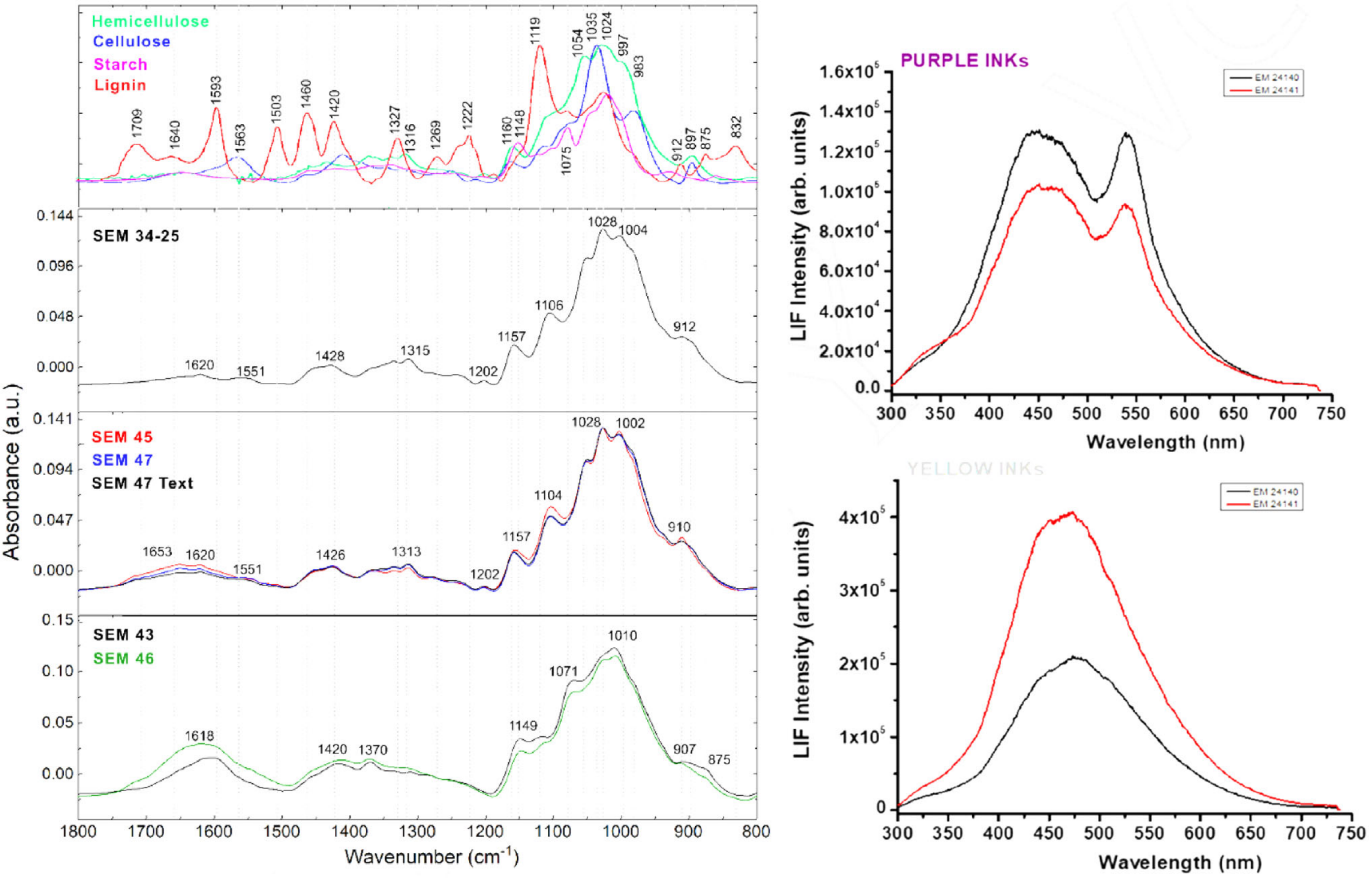
(left) FTIR spectra focusing on the fingerprint region (1800–800 cm^−1^) of red inks from five EM objects, shown alongside reference spectra for cellulose, hemicellulose, lignin [71], and starch [IRUG, sample ICB00021]. (right) LIF spectra of yellow (top) and purple (bottom) inks from objects SEM EM 24 140 and EM 24 141.

Besides the black or dark inks, four additional colored inks—red, yellow, purple, and brown—were identified within the collection. Red ink appears alongside black ink in five objects, while combinations of yellow, purple, or brown inks with black ink were observed in six objects. Regarding the red inks, analysis of the HSI data cubes in the VNIR range (400–1000 nm) showed spectra of the colored inks comparing to the black inks. Raman analysis of red inks allowed the discrimination of families of chromophores produced by both parasitic insects and from vegetal origin. Raman spectra of the red ink (Figure 4) in objects SEM EM 23 134 and SEM EM 24 145 show bands of use of organic dyes such as alizarin crimson [68] as a plant‐driven anthraquinone dye, perhaps madder [68], at strong band at 1480 cm^−1^ associated with stretching C=C, a shoulders of C=C ring stretching at 1462 and 1451 cm^−1^, as well as a C—O fingerprint peak at 1328 cm^−1^, with other characteristic signals at 1292, 1192, 1163, 904, 659, and 480 cm^−1^ [69]. In addition, there are bands associated to alizarin lake [67] with strong C=O stretching mode at 1630 cm^−1^, which is typical for alizarin bound to metal ions, as well as other peaks at 1570, 1532, 1472, 1453, 1358, 1329, 1297, 1190, 1160, 1025, 904, 839, 673, 654, and 486 cm^−1^. Peaks of purpurin lake [67] were also detected in object 24 139 at 1168, 1330, 1388, and 1617 cm^−1^. FTIR spectra (Figure 8a) have confirmed that objects SEM EM 24 145, 24 147, and 23 134–25 share similar spectral characteristics, while samples SEM EM 24 143 and 24 146 form a separate group. In the first group, peaks observed around 1650–1600 cm^−1^ likely correspond to anthraquinone‐based dyes such as alizarin or purpurin. Additional peaks in the regions of 1020–1000 and 700–500 cm^−1^ suggest the presence of lake pigments, most probably formed with metal mordants. The binder in this group is most likely protein‐based, as indicated by the presence of amide I and amide II bands near 1650 and 1550 cm^−1^, respectively. The second group differs from the alizarin/purpurin group. Their spectra are dominated by a peak around 1620 cm^−1^, which perhaps related to aromatic C=C stretching or moisture, without clear amide I–II features. Peaks around 1030–1000 cm^−1^ and in the lower region 700–500 cm‐1 are likely associated with inorganic red lake pigments—possibly iron‐ or manganese‐based, possibly bound with gum Arabic or other carbohydrate‐based materials. The fluorescence emission bands in the 550–650 nm range typically captures the red fluorescence emission of organic compounds, particularly at  ~560–580 nm from madder lake (e.g., alizarin and purpurin); ~590–610 nm suggests alizarin crimson, and shoulders at ~620–650 nm could indicate a complexation with metal ions such as aluminum, calcium, or iron altering the fluorescence shift (Figure 8b). As reported in previous studies and historical resources [69], red organic dyes have been used in Islamic manuscripts since the 9^th^ C, where alizarin was one of the most common organic dyes used in historical Islamic manuscripts. However, the purple and brownish/yellowish ink (gold‐like ink) showed similar Raman bands but with other broad bands at 1550 and 1350 cm^−1^. The FTIR analysis of the black, brown ink in object 24 138 yielded spectrum with bands located at 420, 465, 531, 592, 655, 1002, 1026, 1096, 1157, 1262, 1321, 1377, 1592, 1635, 2915, 3329, and 3396 cm^−1^ that can be attributed to the black and brown inks. In addition, yellow ink showed different bands at 467, 555, 664, 1102, 1159, 1315, 1368, 1424, 1620, 2892, and 3331 cm^−1^. Therefore, the shades of the brown and yellow inks may be produced by another component of the ink recipe not apparent on the spectrum [70]. The laser‐induced fluorescence spectra of the yellow ink in SEM EM 24 140 and 24 141 samples consist of a broad emission band with a maximum at 475 nm, similar to that observed for the paper substrates, without any additional specific bands. However, the fluorescence emission of the same samples but from the purple areas show similar emission spectrum as for black ink with an extra band at 550 nm, indicating that the purple color is probably a product of mixing black and red inks (Figure 8b). LIBS spectrum from red ink in sample SEM EM 24 143 show qualitatively similar composition as for black ink in the same sample with quantitative differences (Figure 5c). This would indicate that the dyes responsible for the red color could be of organic composition as already indicated by LIF (alizarin and/or purpurin).

Comparing the selected objects based on the textual and decorative evidence, it has become clear that there is a specific grouping, purposes, and decoration that these amulets and talismans share. It combines Qur’anic orthodoxy with local folklore or magic, typically in West African and Maghrebi traditions where Islam coexist with indigenous spirituality [1]. The studied objects were chosen to have different support papers, sizes, decorations, inks, writing styles and topics, so they may belong to different scribal schools or workshops. The objects could be classified into amuletic or talismanic nature based on the existence of texts or symbols or drawings which is likely from West Africa regions where Islam blended with local artistic and spiritual practices. Such objects were typically created by Sufi healers (*marabouts*) or Islamic scribes (‘*ulama*). The use of written documents in connection with protection or magic is widespread throughout the Muslim world [72]. With the introduction of Islam in Central and West Africa, the power of oral learning was gradually replaced by the power of Arabic script and intermingled with the use of African traditional objects for the practise of protection and magic [1]. Therefore, this practise goes beyond the African context where there is evidence of the use of pictorial art as the main tool for making amulets and talismans in the early and middle Islamic period [73]. There are widespread traditions (and even alchemical rituals) among Muslims related to supernatural powers (e.g., jinns and the evil eye). The belief in amulets continued across Islamic regions for centuries, with a notable preference for clear verbal amulets derived from the Qur’an and Sunnah, rather than imagery‐based forms. In contrast, other regions demonstrated a stronger reliance on symbolic amulets incorporating numbers, magical squares, and talismanic figures [13]. Furthermore, the verbal amulet tradition was not confined to Islamic contexts; it spread beyond, as evidenced in the Balkan Byzantine culture, where Qur’anic invocations were adapted into local practices [74]. This comparative perspective significantly enhances the understanding of the uniqueness and representativeness of the samples in this study, and we will integrate this observation more explicitly into our discussion. When we look at calligraphy in this collection, we can sort the studied manuscripts into three main categories: i) rudimentary with the use of local coping, plain script, minimal layout structure (e.g., SEM EM 23 134–10); intermediate with semiprofessional script, some decorative elements, and structured layout (e.g., SEM EM 24 143–44); and exemplary works with scholar commission, rich illumination, deluxe materials, and high artistry (e.g., SEM EM 24 138–42) [75]. The objects vary in quality and have a certain structure and specific details that empower them with protection or magic: the way they address God, the choice of Qur’anic verses, and the graphic, numerological, and textual elements; some of the protective texts contain magic squares using either letters or numbers or a combination that are understood to have ‘inherent’ occult power based on the geometric forms. The use of decorative elements, geometric designs, vegetal devices, and even color as frames and text divisions strongly suggests that such enhancements were meant to appeal to a specific clientele for the amulets/talismans. The reason for the inclusion of such features could be to increase the visual appeal of the item to a prospective buyer or user. These manuscripts utilize the Kufic script, particularly within the Maghrebi calligraphic tradition. The exemplary works are often seen as the pinnacle of Kufic style, while the others show various derivatives with their own unique ways of forming letters. The scribes who created the rudimentary manuscripts often learned Arabic literacy just before or while they were writing, which leads to some noticeable technical flaws. On the other hand, the exemplary manuscripts reflect a deep understanding of classical Kufic principles. The master calligraphers behind these works display remarkable skill in producing Islamic manuscripts, showcasing advanced letter formation, a sophisticated use of color, and intricate design techniques. One striking feature of their work is the consistent use of red ink to highlight different forms of the divine name “*Allâh*,” a practice commonly found in Qur’anic and religious manuscripts. The contents of these manuscripts cover a wide array of topics, including verses and chapters from the Qur’an—some even arranged in magic squares. They also feature scribal invocations, devotional phrases, and liturgical texts. A notable example of this is the Sufi litanies, which sometimes serve as protective charms, marked by repetitive prayers. For instance, manuscript SEM EM 24 147 includes repeated inscriptions of “Ya *Allâh*,” while manuscript SEM EM 24 146 weaves in similar repetitive elements alongside Qur’anic excerpts, divine names, references to angels, mentions of prophets, caliphal names, and specific litanies—sometimes written in mirror script for added depth [76]. The color schemes, decorative elements, and structural designs of these manuscripts reveal significant influences from the regional cultures. This local esthetic adaptation helps to explain the similarities we observe between Islamic manuscripts and their non‐Islamic and non‐Arabic counterparts produced in the same areas. The Moroccan calligraphy was probably used in the objects [74, 77] which evolved from Kufi script as typical to Western African world and Andalusia in term of the style of rounded letters, extended horizontal strokes, pronounced loops, and deep curves, as well as the writing style of letter *phaa* (ف) (where the dot is placed under the letter) while the final letter, e.g., Nūn (ن) has an open, extended tail, unlike the closed‐loop version seen in eastern calligraphic styles. In addition, there is a use of thick strokes in certain letters often contrasting with thin lines and use of golden color or highlight of important words or letters. The differences in calligraphic execution suggest varying levels of expertise between the manuscripts. Some appear the work of a local Muslim scribe with basic Arabic knowledge, while others appear to be the product of a seasoned calligrapher, potentially an “ustadh” or professor who had refined their skills over years of dedicated practice and instruction. However, most Muslims agree that Islam forbids the invocation of jinn or the use of sorcery, as Islamic theological tradition emphasizes the belief that Muslims should place their trust solely in *Allâh*, for protection from sorcery and malevolent spiritual forces, rather than resorting to talismans, amulets, or other means of safeguarding. The authentic teachings of Islam discourage the use of figurative amulets, which were common in ancient cultures, and instead recommend the utilization of verbal charms derived from the Qur’an and the sayings of the Prophet Muhammad [78]. Reliance on traditional religious healers is most widespread among Muslims in sub‐Saharan Africa, with the majority of people, for example in Chad and Mali (as well as in the Balkans due to the influence of Ottoman Empire), reported having turned to traditional healers to cure a sick person [6]. In conservative Islamic realm, the talisman is seen as bordering on *shirk* (polytheism—the association of partners with *Allâh*) when belief in its power overshadows reliance on *Allâh*. Therefore, there is a generalization of the prohibition of magical talismans, likewise amulets, if they are mixed with practises that are considered non‐Islamic such as animism. This depends on the talismanic purposes and intentions of the Muslim and non‐Muslim talisman makers, who are individuals such as healers or diviners with a reputation for mystical knowledge [70, 79]. It is more common for Muslims to wear or display verses from the Qur’an in their homes than to own talismans to ward off the evil eye. Amulets are generally accepted in the traditions Muslim communities if they contain Qur’anic verses or prayers, as they are seen as extension of the *du’a* (supplications) for protection. Therefore, amulets are used in rural and urban areas by people of all social classes and are often made by Islamic scholars who are trusted for their religious knowledge. Amulets or talismans gain their power firstly through their customized shape and form and secondly through their application by simply being worn or placed, while others can only be activated by reading the text written on them. The specific arrangement of texts and elements on the amulet or talisman is achieved by carving, modeling, or assembling the materials into symbolic shapes or by inscriptions [49]. The ritual procedures for activating the powers associated with the meaning and multiple uses of amulets and talismans consist, for example, of uttering or reciting certain religious or magical formulae in conjunction with certain features (inscriptions, symbols) and associating the object with a source of power such as a sacred place or person, depending on the nature of the text (medicinal or religious) and the geographical, historical, and socio‐intellectual context in which they were made and acquired, particularly in Africa. Even if the terminology is controversial, an amulet (primarily protective and infused with blessing‐ *baraka*) or a talisman (primarily harmful) is an object endowed with a power that manifests itself in many cultures [80]. The objects were subject multiple analyses to identify the papers, inks, coloring materials, and paper used by the handcrafting scribes and illuminators and to assess whether there were changes of pigments and recipes, use of sizing, and local paper fibers. The reconstitution of watermarks occurring in the inner margins of tightly sewn manuscripts, the time needed for categorizing the watermarks by similarity, and, above all, their presence, mostly limited to European manually produced paper up to 19^th^ century. The fiber furnish analysis showed the use of softwood pulp and straw. Conifers such as pine are rich in long, strong cellulose fibers, commonly found in mechanical or chemical wood pulps. This is typical to European‐style industrial paper making possibly late nineteenth to twentieth century where paper or pulp was imported from colonial era paper mills that operate in African regions. This pulp is less likely to be from traditional Islamic papermaking which typically used non‐wood fibers like flax, hemp, or cotton rags through the centuries until 19th century. Straw is typically short‐fibered and more brittle used mainly in local paper production. The use of mixed furnish (wood pulp and straw) strongly suggests a post‐colonial period of 19^th^ C where local Qur’anic schools and Islamic educational centers tried to localize the papermaking using the locally available resources and economization of the paper industry. However, since the 19^th^ C, European paper was imported to Islamic countries. This may indicate that these sheets are not subject to sheet‐by‐sheet quality control or 24 140 is not made of the same paper mill. Softwood pulps are more acidic and prone to oxidative degradation over time (e.g., yellowing and embrittlement), while straw pulp also contains lignin and hemicellulose, making it even more vulnerable to chemical degradation and necessitate conservation treatments such as deacidification with proper environmental control (e.g., temperature and humidity) to slow down the acid hydrolysis. The investigations showed that the tested objects were very acidic. It is well known that acidity plays a very important role in paper and ink degradation; the more acidic the paper is, the faster the degradation proceeds.

The analyzed inks include black, dark brown, pale brown, red, purple, and yellow inks. The results revealed a small palette of coloring materials with application of different shades of color including mainly iron gall and dyes such as alizarin crimson, alizarin lake, and purpurin lake which seems to have been used partly for its rich lustrous finish [67]. The colored inks were partially used for specific words or motives which might be used for medical purposes in the Western African Islamic manuscripts [81]. The HSI maps were used for characterization of inks and coloring materials and as a guide to select zones of interest and minimize the spots for investigation by the elemental and spectroscopic analysis techniques. The first screening using HSI ordinary color pictures and the cases of multichannel acquisitions in the visible and non‐visible ranges allowed a detailed documentation regarding the overall state of preservation of the paper and inks including absence of previous conservation treatments and polishing marks and existence of initial writing where the iron gall inks were originally used without application of corrections or latter interventions or corrections. The assessment of HSI imaging supported the enhancement of the ink for comparison and identification of inks based on the color and image features [82]. The black and colored inks showed different optical characteristics and spectral profiles with no ink loss. HSI also documented the cracks, abrasion, and scratches with no fading of the initial inscriptions. Raman spectroscopy has been demonstrated to be very useful in the identification of the black and colored inks. Spectral similarity was detected in Raman analysis of black inks except two objects where a mixed ink of iron gal and carbon‐based inks were recorded. Raman spectroscopy allowed rapid and unambiguous in situ identification of the majority of inks and dyes used by the calligraphers. All the objects showed iron gall ink Raman spectra, except object 24 140 where characteristic bands carbon black ink was presented along with the IGI bands of which a mixed iron gall ink/carbon‐based ink was probably used in this object. All LIF spectra of the analyzed objects consist of a broad feature in the 300–750 nm region, although the different samples display special characteristics that are associated with their nature of the paper substrate and inks composition. LIBS analysis of a few ink stains allowed an in‐depth view within the chemical fingerprint of the original iron gall ink. Fe, Mg, Ca, Al, and Na were perhaps related to the vitriol recipe used for the iron gall ink preparation. Lines of the main elements (Fe, Ca, Mg, and Na) were observed in the spectra of all analyzed black inks with different intensities revealing different contributions of these elements according to the used recipes. In LIBS analysis, C, CN, and C_2_ can represent the major organic compound of the ink, e.g., tannins, arabic gum, or paper, while Fe is the main ingredient of IGI from ferrous sulfate, and Mg is also a trace element often present with iron ores. The presence of Al and K elements could be from alum which was likely added to ink as a mordant or pH adjuster.

## 4 Conclusions

The Slovene Ethnographic Museum (SEM) holds a valuable amuletic and talismanic Islamic‐African paper manuscripts as a unique oriental collection in Slovenia. These selected objects offer valuable insights into the cultural and artistic practises of the regions where they originate. This study offers the first integrated archaeometric and contextual investigation of a group of West African Islamic amulets and talismans, revealing their material composition, scribal practices, and cultural functions. By combining HSI, Raman, FTIR, LIF, and LIBS with philological and paleographic analyses, we were able to identify a limited range of inks, including iron gall, carbon black and anthraquinone‐based dyes such as alizarin and purpurin, which were often used selectively for symbolic or religious purposes. Spectral analyses also distinguished between organic and inorganic lake pigments and showed that the recipes were based on both local resources and imported European paper materials, highlighting the coexistence of indigenous practises and colonial‐era materials. The results confirm that the analyzed manuscripts were not only functional texts but also objects with ritual and protective significance. Their varying calligraphic execution, ranging from rudimentary to masterful styles derived from kufic, reflects different scribal schools and levels of training. The integration of Qur’anic verses with geometric motifs, magic squares, and decorative color schemes illustrates the blending of orthodox Islamic traditions with local esoteric and spiritual practises. From a conservation perspective, the evidence of acidic wood and straw pulp alongside the decomposition signatures of iron gall inks emphasizes the urgent need for preventative measures such as deacidification and controlled storage in the environment. Furthermore, the ability to differentiate ink compositions and trace element additions offers valuable insights into historical recipes, scribal interventions, and potential re‐inking practises, while also providing information for targeted conservation strategies.

Culturally, these objects are an example of how Islamic manuscript traditions in West Africa were adapted to meet community needs for protection, healing, and devotion. Their study enriches our understanding of the materiality and spirituality of Islamic heritage while contributing to a wider comparative debate on the use of amulets and talismans in different regions and faiths. The results will enable informed conservation decision making by the museum staff and allow the curators to plan the display of the collection to the public for the first time with understanding of the textual, technology, provenance, and state of conservation of the paper collection. The research will undoubtedly influence collection management in the Slovene Ethnographic Museum and more widely the Slovenian museums. The overall results are in complement to each other providing advantages of the use of multiple non‐destructive and destructive techniques. Further in‐depth research will focus on understanding the degradation of inks and paper supports as well as on collection management and the requirements of preventive conservation for temporary or permanent exhibitions.

## Supporting Information

Additional supporting information can be found online in the Supporting Information Section. **Supporting Fig. S1:** LIF spectra of paper substates from different samples upon laser excitation at 266 nm (top is LIF spectra as collected using gate delay and width of 0 and 3μs, respectively; bottom: normalized LIF spectra for comparison both LIF spectra correspond to the accumulation of 15 measurements). **Supporting Fig. S2:** FTIR spectra of black inks collected from 12 EM objects, including samples from both drawing and text. The spectra shows characteristic features associated with iron gall ink (IGI) components. For comparison, two, IGI reference spectra from the historic HSLL collection are also included. **Supporting Table S1:** The selected EM objects for analysis with ID and dimensions (L: length and W: width). **Supporting Table S2:** The 12 EM Objects under transmitted light showing the detected watermarks in objects (SEM EM 23 134‐25; SEM EM 24 138; SEM EM 24 139; SEM EM 24 138; SEM EM 24 141; SEM EM 24 142 and SEM EM 24 147). **Supporting Table S3:** False colour HSI images of the 12 EM objects, generated using principal component Analaysis (PCA) in the SWIR spectral range (one side). **Supporting Table S4:** Results of the spectral angel Mapper (SAM) analysis applied to SWIR hyperspectral images of the 12 EM objects (one side). **Supporting Table S5:** HSI images of 12 EM objects at 1700 nm (one side). **Supporting Table S6:** Graphs of raman spectra of characteristic bands of black Iron Gall ink (IGI) of the selected EM objects. **Supporting Table S7:** Raman spectra of characteristic bands of colored inks of the selected objects. **S** **upporting Table S8:** LIF graphs of the selected objects. **Supporting Table S9:** LIBS Graphs of the selected objects. **Supporting Table S10:** The hyperspectral imaging system's acquisition parameters.

## Funding

This work was supported by EU Horizon IPERION HS project (Integrated Platform for the European Research Infrastructure on Heritage Science); Slovenian node of the European Research Infrastructure for Heritage Science (infrastructure program I0‐E012 of the Slovenian Research and Innovation Agency); Programme Group N‐DAD (P1‐0447) at the Faculty of Chemistry and Chemical Technology at the University of Ljubljana (Slovenia); 20‐RSUL.SLOVENILE.129 ARIS‐RSF project) at the Faculty of Chemistry and Chemical Technology at the University of Ljubljana (Slovenia); ARRS WEAVE project (ABC, N1‐0271) at the Faculty of Chemistry and Chemical Technology at the University of Ljubljana (Slovenia); ARRS WEAVE project (SafeSilk, N1‐250) at the Faculty of Chemistry and Chemical Technology at the University of Ljubljana (Slovenia); PID2022‐137017OB‐I00 project from MCIN/AEI/10.13039/501100011033/FEDER, UE; TEC Heritage‐CM (TEC‐2024/TEC‐39) project from Regional Government of Madrid; “Open Heritage and Research and Society” (PTI‐PAIS) project from CSIC Interdisciplinary Platform.

## Conflicts of Interest

The authors declare no conflicts of interest.

## Supporting information

Supplementary Material

## Data Availability

The data that support the findings of this study are available from the corresponding author upon reasonable request.
