# Supplementary material for: Multianalytical Study of Amuletic and Talismanic Islamic‐African Paper Manuscripts in the Slovene Ethnographic Museum
Source: Chempluschem. 2025 Nov 20;90(12):e202500433. doi: 10.1002/cplu.202500433 (PMC12701302; doi:10.1002/cplu.202500433)
Supplement: Supplementary file 1 — Supplementary Material [file CPLU-90-e202500433-s001.pdf]

Supplementary Materials

Multi-Analytical Study of Amuletic and Talismanic Islamic-African Paper Manuscripts in the Slovene Ethnographic Museum

Abdelrazek Elnaggar[a,b*], Hend Mahgoub[a], Laura Maestro-Guijarro[c], Ana Crespo Ibáñez[d], Paula María Carmona-Quiroga[c], Santiago Sánchez-Cortés[d], Žiga Rehar[e], Gregor Kos[e], Ahmed Ameen[f], Marko Frelih[e], Matija Strlič[a,g], Mohamed Oujja[c], Marta Castillejo[c]

[a] A. Elnaggar, H. Mahgoub, M. Strlič
Heritage Science Lab Ljubljana, Faculty of Chemistry and Chemical Technology, University of Ljubljana
Večna pot 113, 1000 Ljubljana, Slovenia
E-mail: abdelrazek.elnaggar@fkkt.uni-lj.si

[b] A. Elnaggar
Archaeological Science and Excavation Department, Faculty of Archaeology
Ain Shams University, 11566 Abbasia, Egypt

[c] L. Maestro-Guijarro, P. María Carmona-Quiroga, M. Oujja, M. Castillejo
Instituto de Química Física Blas Cabrera, IQF-CSIC, Madrid, 28006, Spain

[d] A. Crespo Ibáñez, S. Sánchez-Cortés
Instituto de Estructura de la Materia, IEM-CSIC, Madrid, 28006, Spain

[e] Žiga Rehar, Gregor Kos, Marko Frelih
The Slovene Ethnographic Museum (SEM), Ljubljana, 1000, Slovenia

[f] A. Ameen
University of Sharjah, Sharjah, 27272, United Arab Emirates

[g] M. Strlič
UCL Institute for Sustainable Heritage, University College London
14 Upper Woburn Pl, London WC1H 0NN, United Kingdom

**Table 1.** The selected EM objects for analysis with ID and dimensions (L: length and W: width)		
**SEM EM 23134-10 (Two sides)** 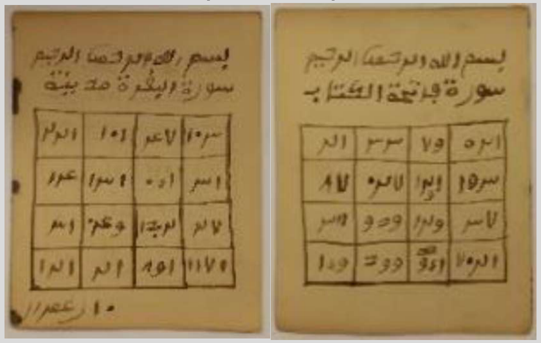 ~11 (L) x 8.7 cm (W)	**SEM EM 23134**-25 **(Two sides)** 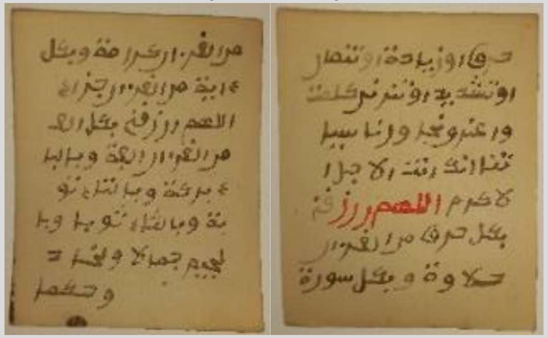 ~10.5 (L) x 8.5 cm (W)	**SEM EM 24138 (Two sides)** 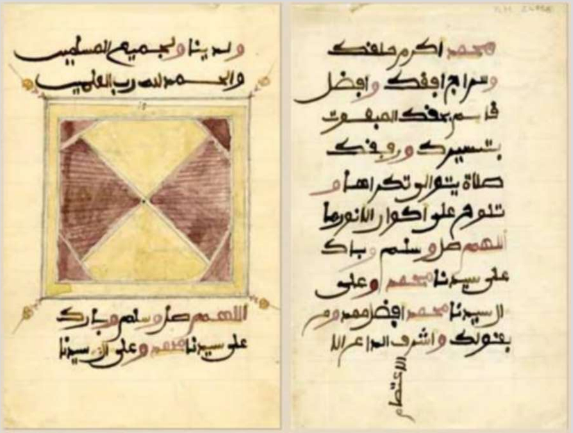 ~16.5 (L) x 10 cm (W)
**SEM EM 24139 (Two sides)** 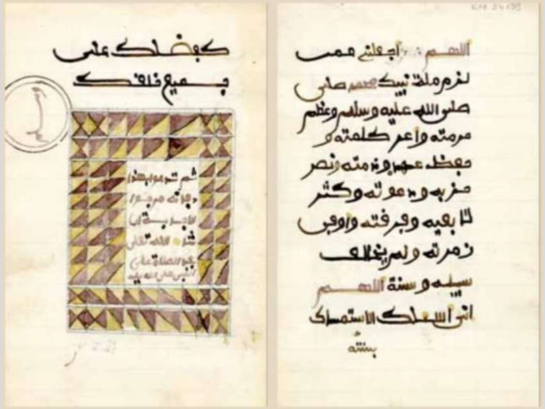 ~16.5 (L) x 10 cm (W)	**SEM EM 24140 (Two sides)** 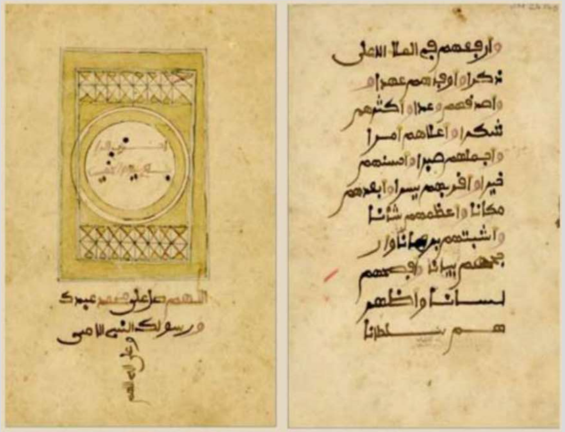 ~16.5 (L) x 10 cm (W)	**SEM EM 24141 (Two sides)** 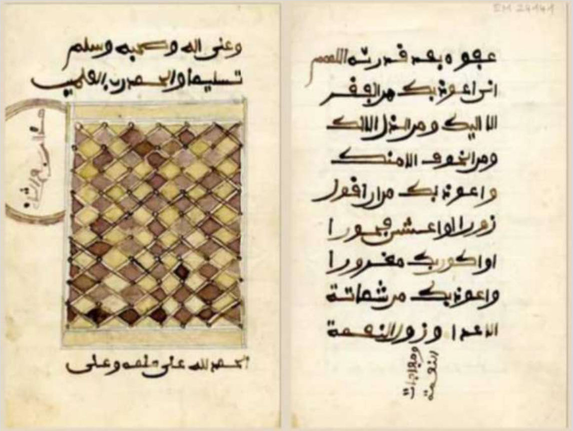 ~16.5 (L) x 10 cm (W)

**SEM EM 24142**<br>**(Two sides)**<br>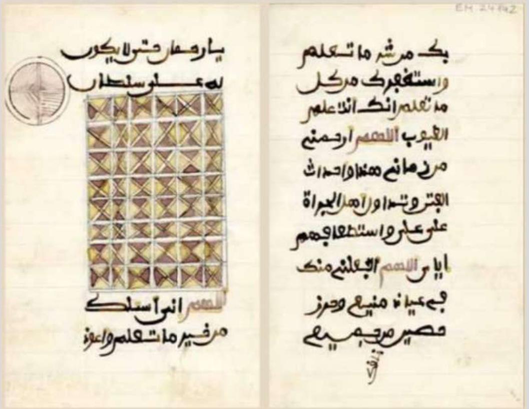<br>~16.5 (L) x 10 cm (W)	**SEM EM 24143**<br>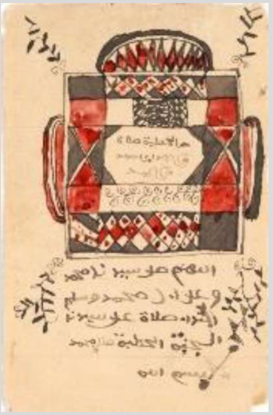<br>~16.5 (L) x 10 cm (W)	**SEM EM 24144**<br>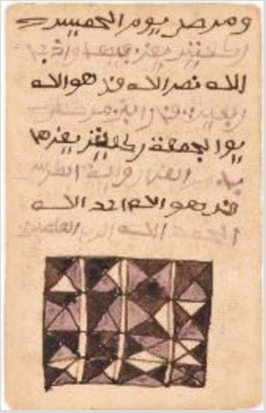<br>~16.5 (L) x 10 cm (W)
**SEM EM 24145**<br>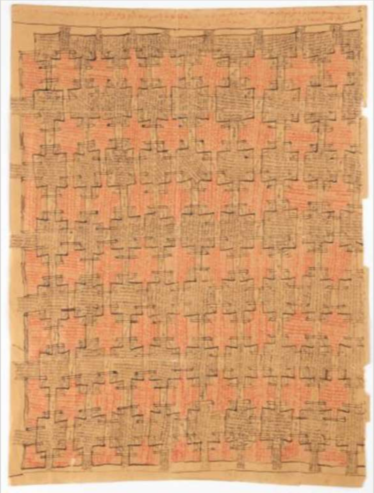<br>~52 (L) x 39 cm (W)	**SEM EM 24146**<br>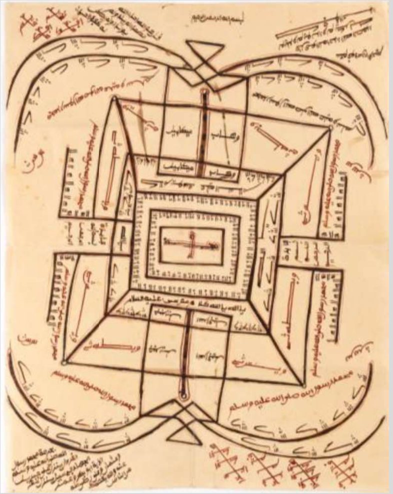<br>~41 (L) x 32 cm (W)	**SEM EM 24147**<br>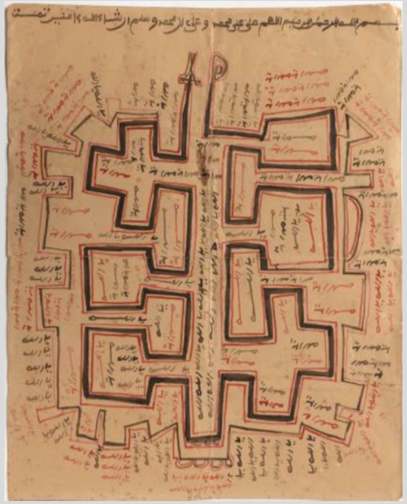<br>41 (L) x 32 cm (W)

Table 2. The 12 EM objects under transmitted light showing the detected watermarks in objects (SEM EM 23134-25; SEM EM 24138; SEM EM 24139; SEM EM 24138; SEM EM 24141; SEM EM 24142; and SEM EM 24147)

**SEM EM 23134-10**	**SEM EM 23134-25**	**SEM EM 24138**
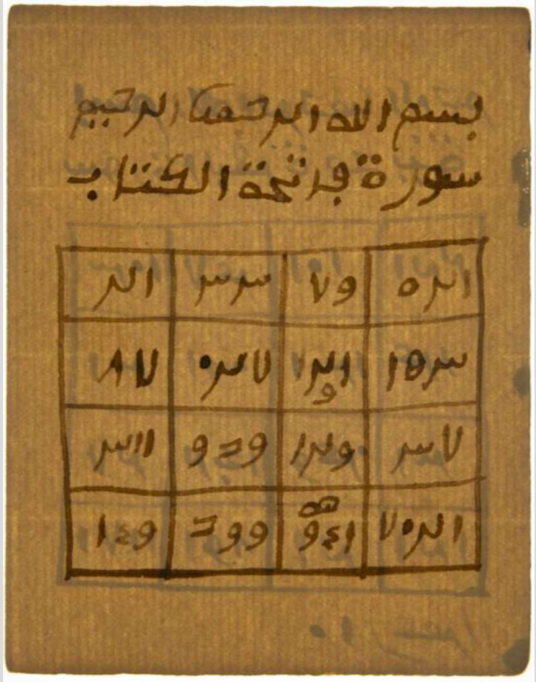	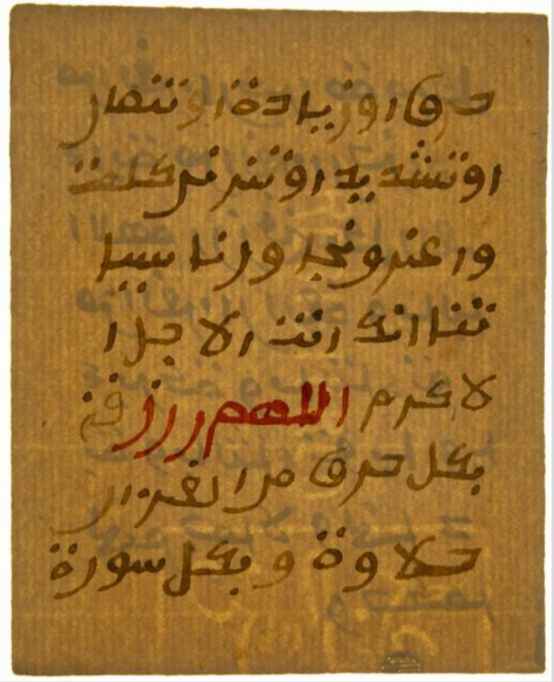	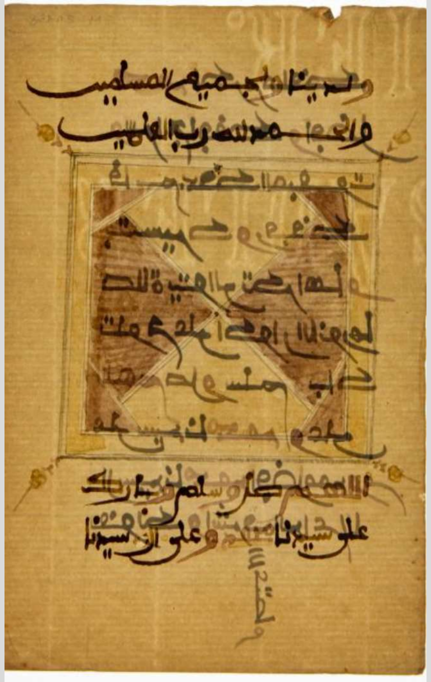
**SEM EM 24139**	**SEM EM 24140**	**SEM EM 24141**

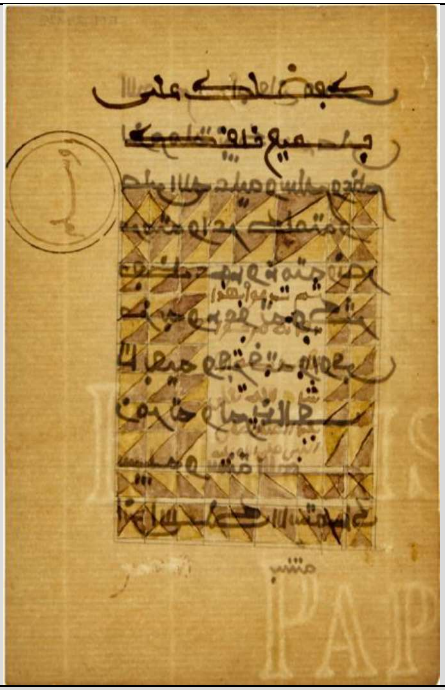	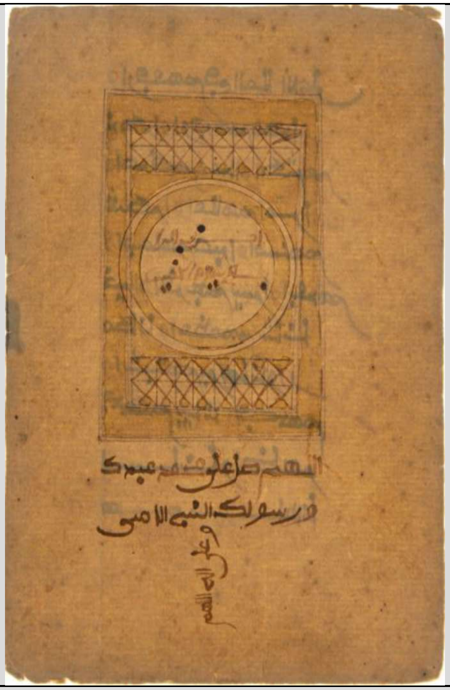	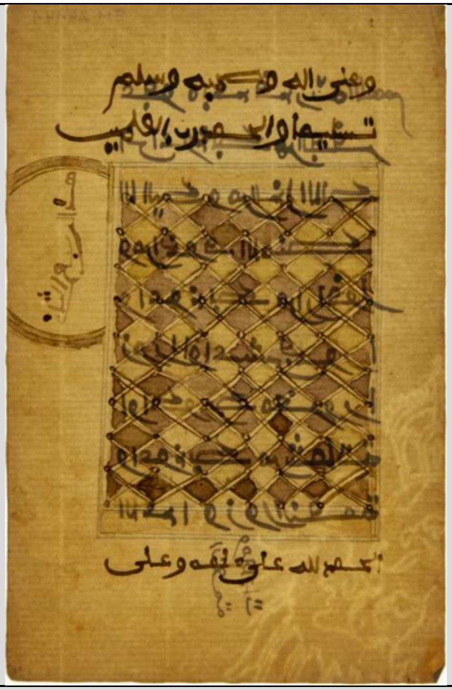
**SEM EM 24142**	**SEM EM 24143**	**SEM EM 24144**

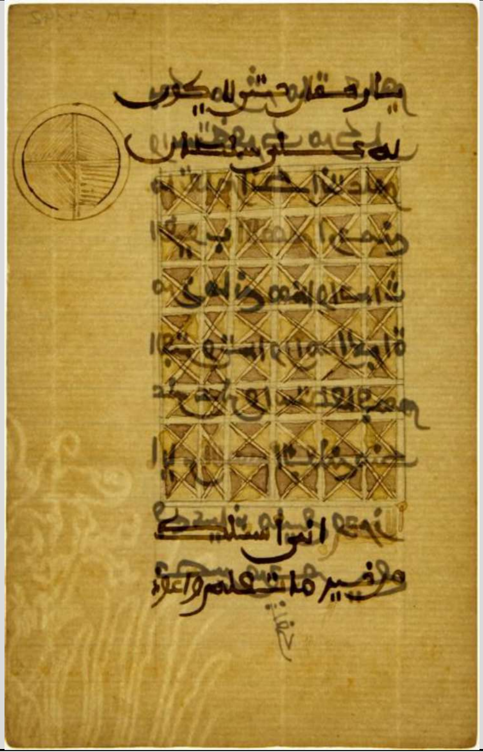	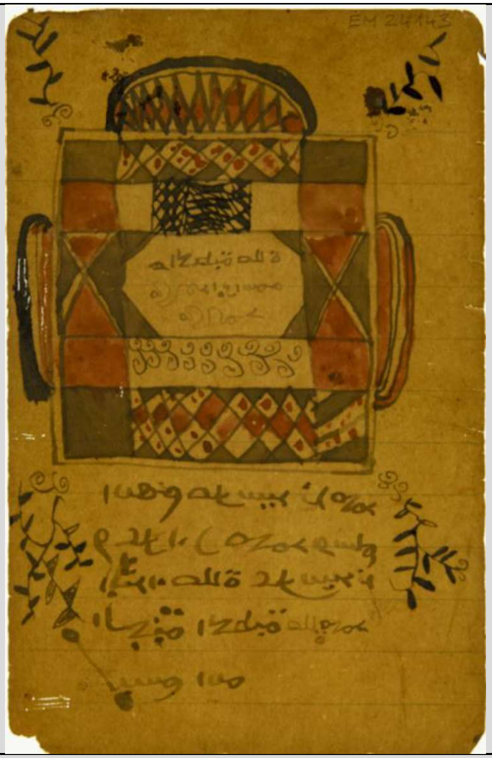	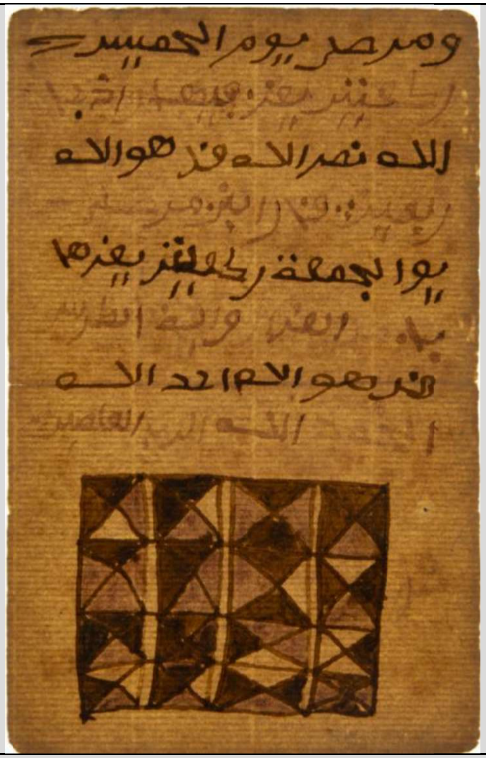
**SEM EM 24145**	**SEM EM 24146**	**SEM EM 24147**

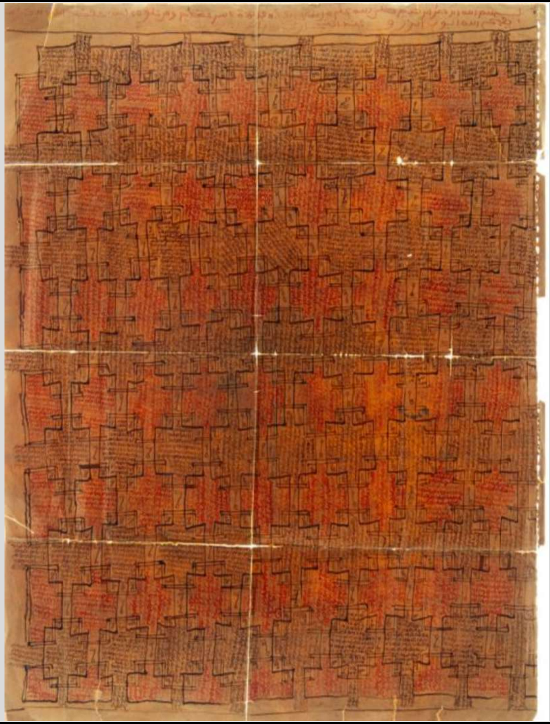

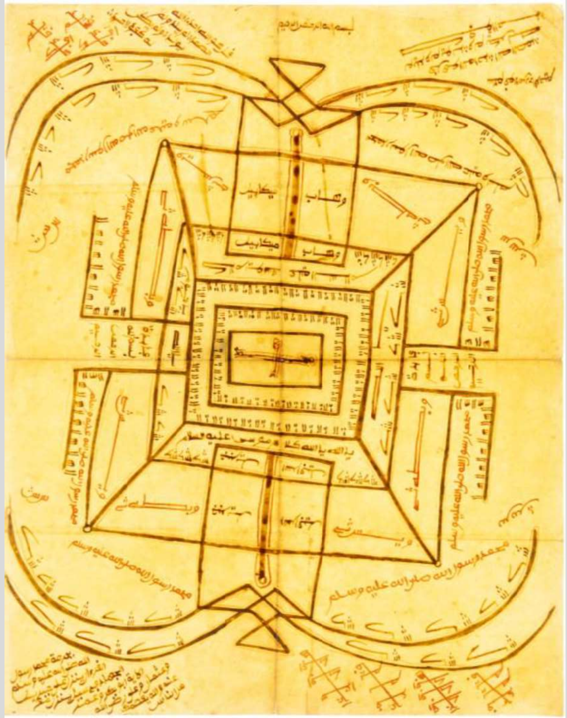

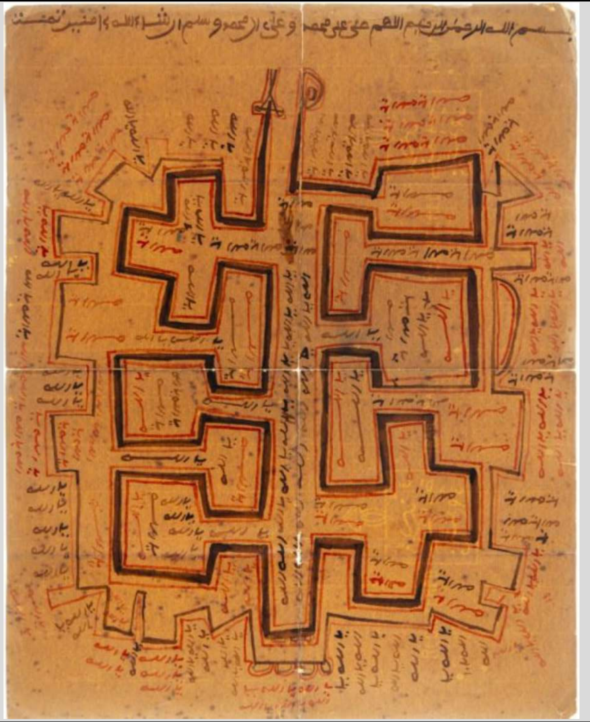

Table 3. False-colour HSI images of the 12 EM objects, generated using Principal Component Analysis (PCA) in the SWIR spectral range. (one side)

**SEM EM 23134-10** 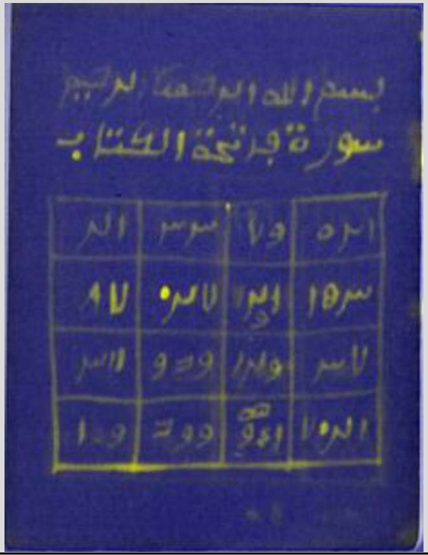	**SEM EM 23134-25** 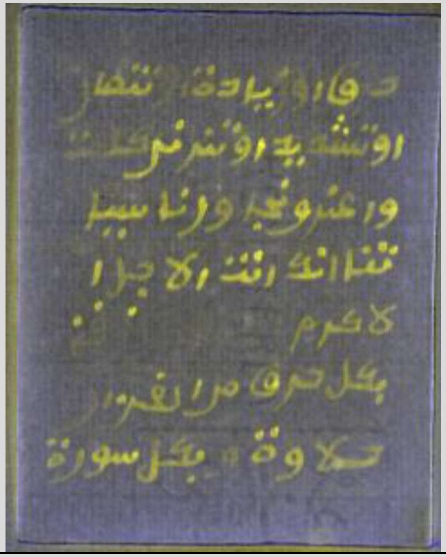	**SEM EM 24138** 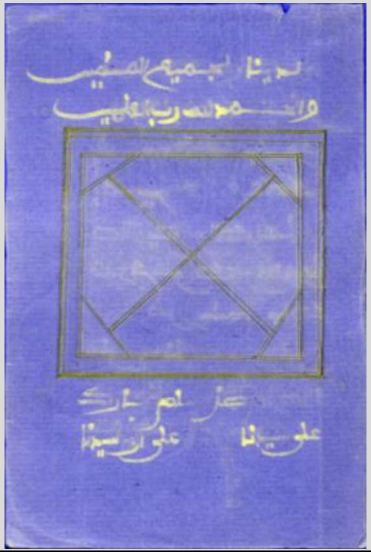
**SEM EM 24139** 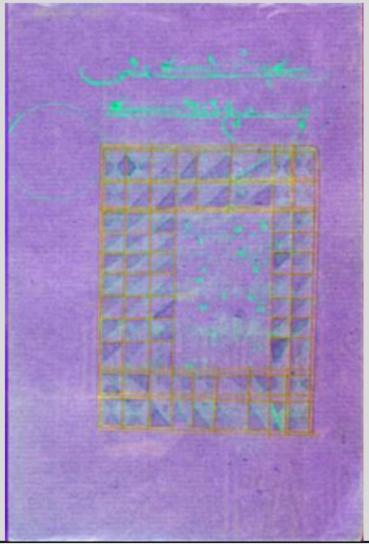	**SEM EM 24140** 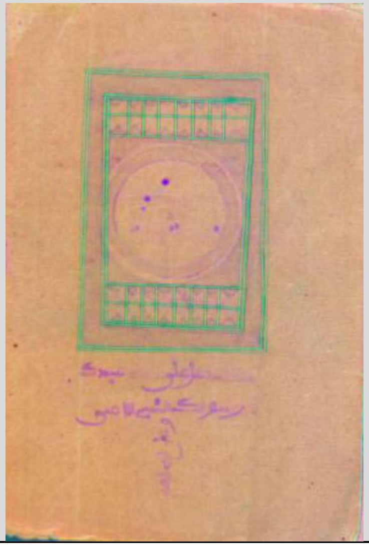	**SEM EM 24141** 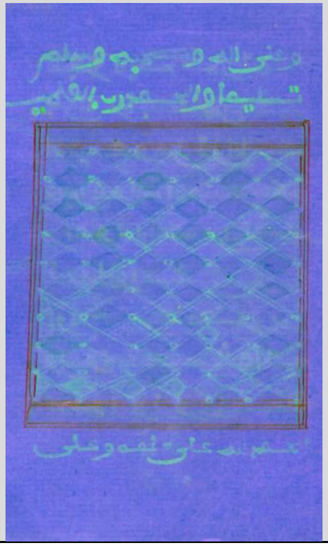

SEM EM 24142	SEM EM 24143	SEM EM 24144
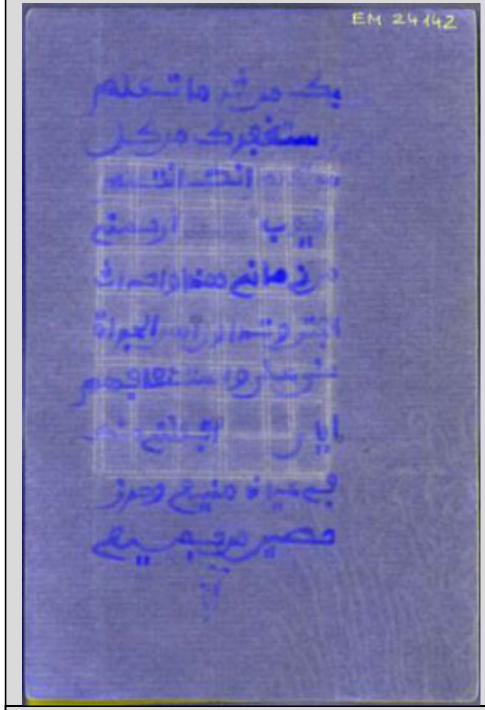	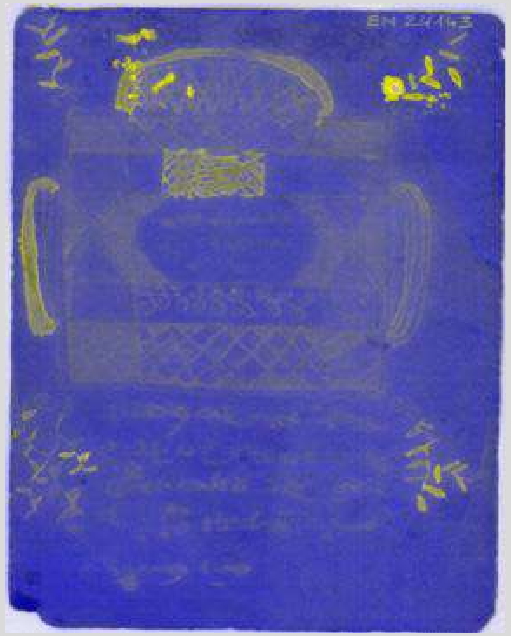	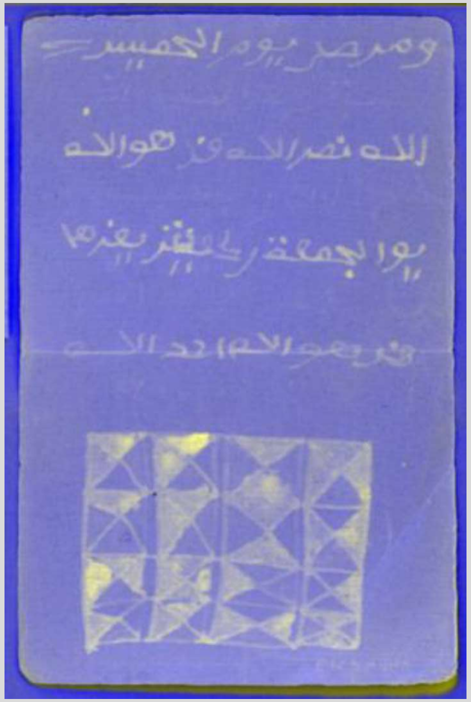
SEM EM 24145	SEM EM 24146	SEM EM 24147

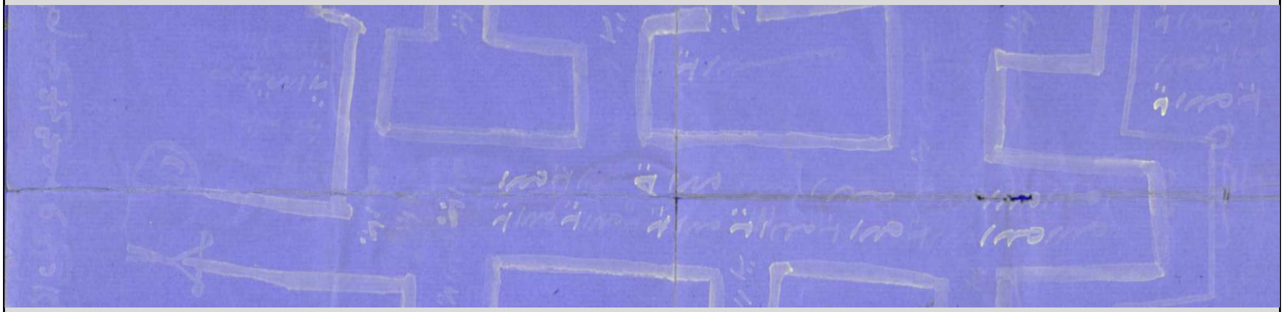

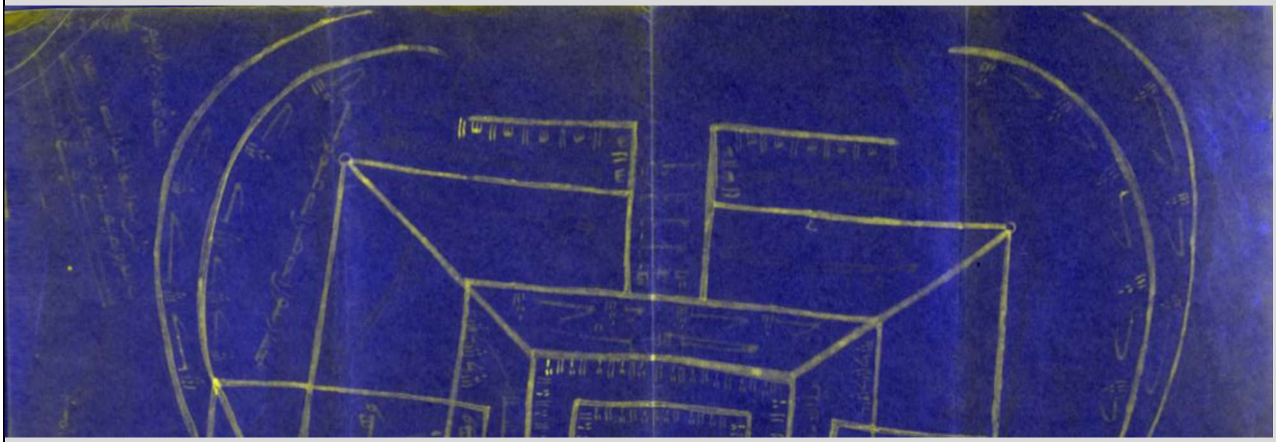

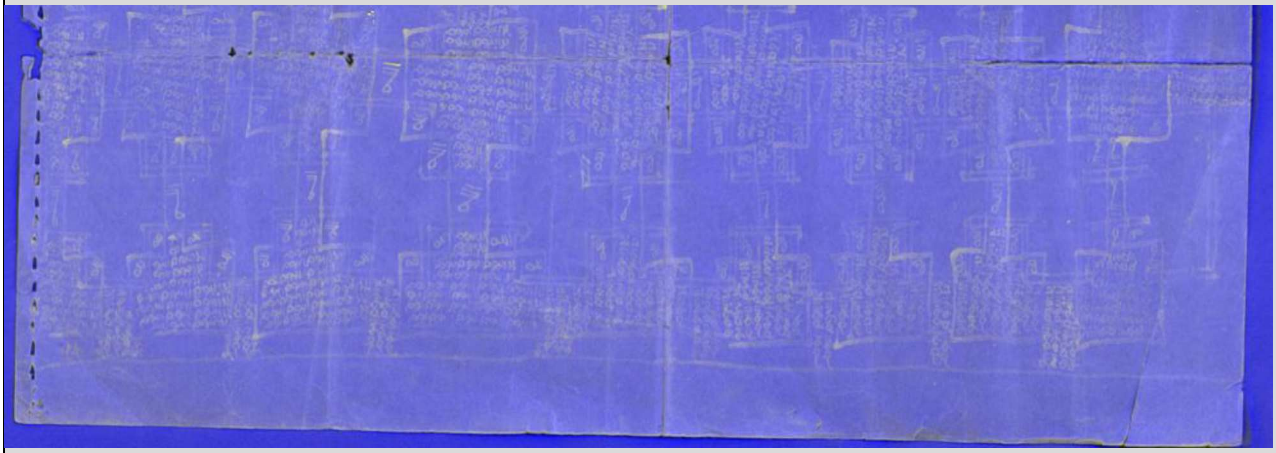

**Table 4.** Results of Spectral Angle Mapper (SAM) analysis applied to SWIR hyperspectral images of the 12 EM objects. (one side)	
**SEM EM 24139**	**SEM EM 24138**
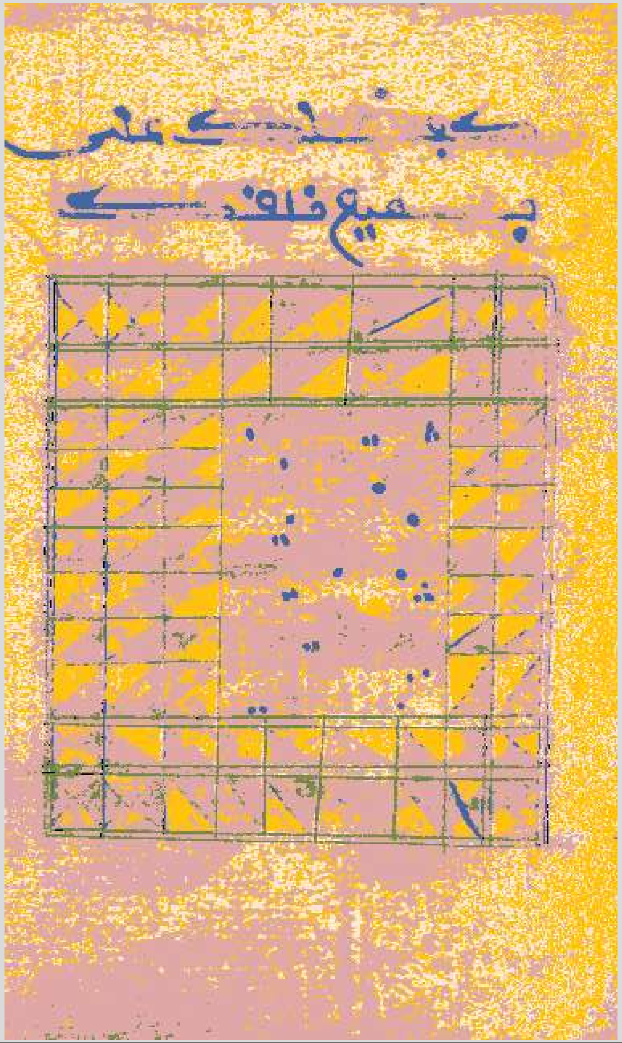	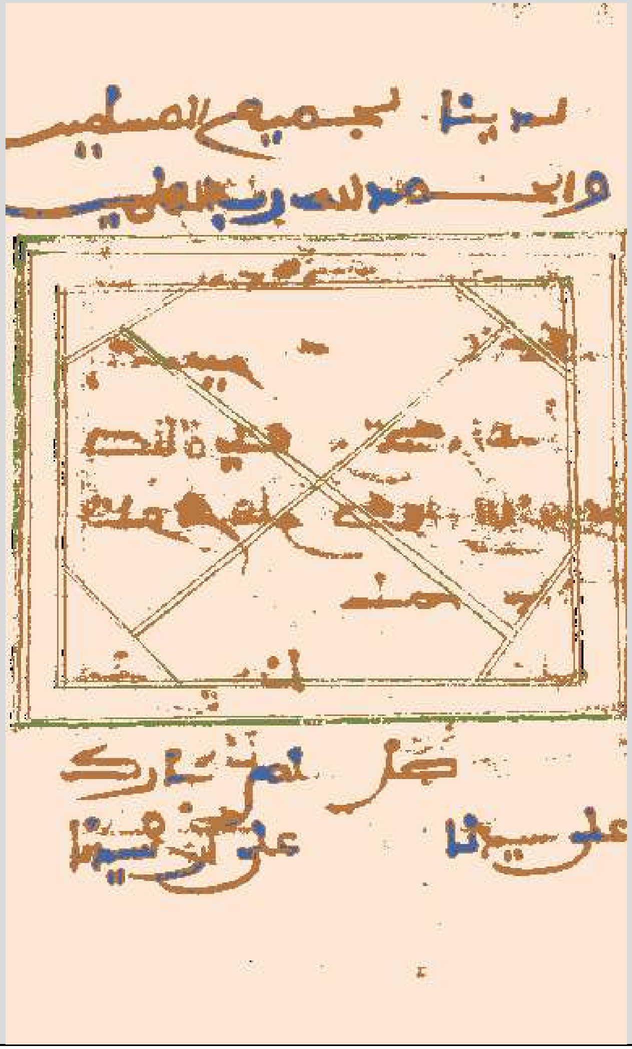

SEM EM 24140	SEM EM 24141
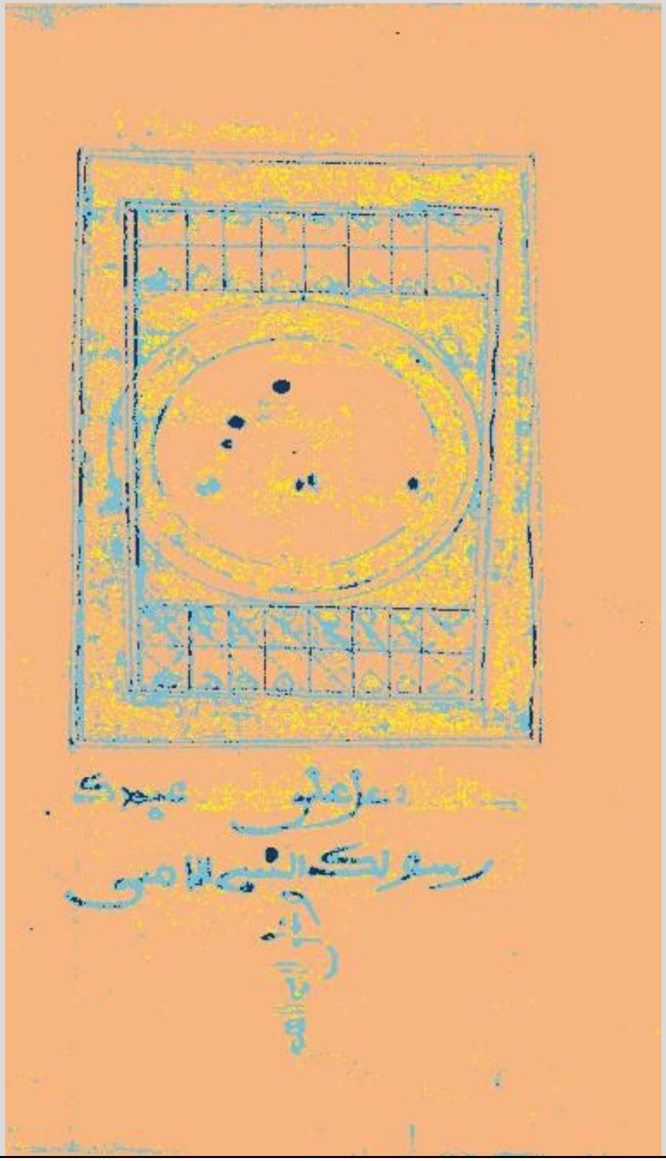	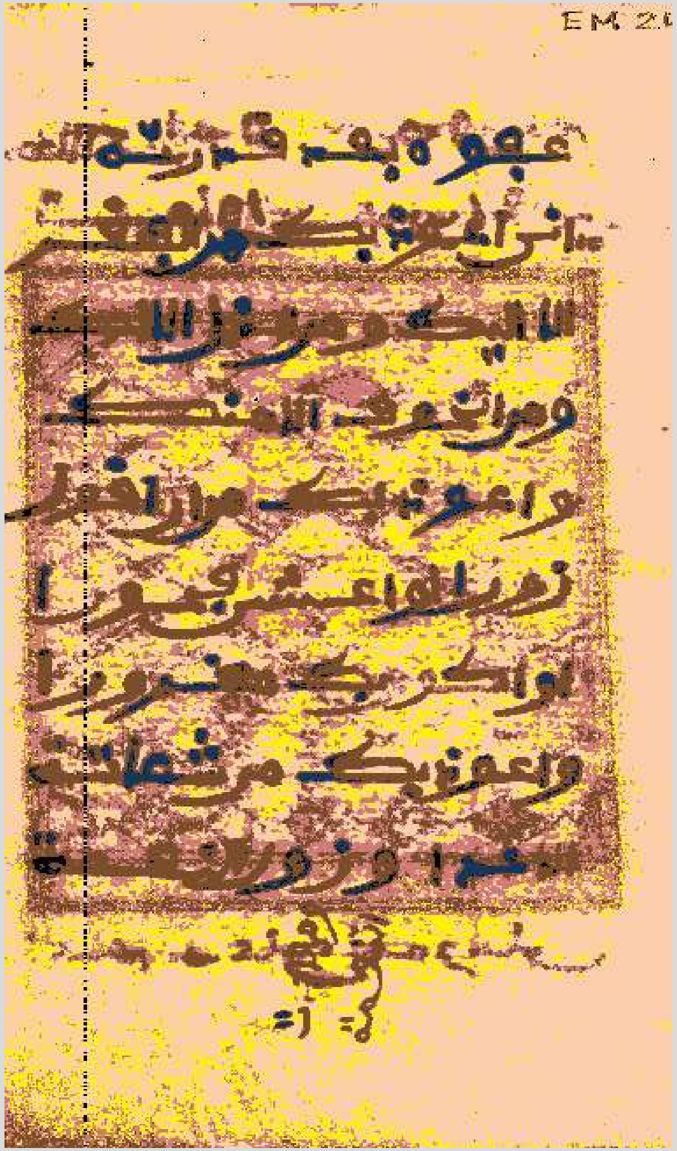

Table 5. HSI images of 12 EM objects at 1700 nm (one side)

**SEM EM 23134-10**	**SEM EM 23134**-25	**SEM EM 24138**
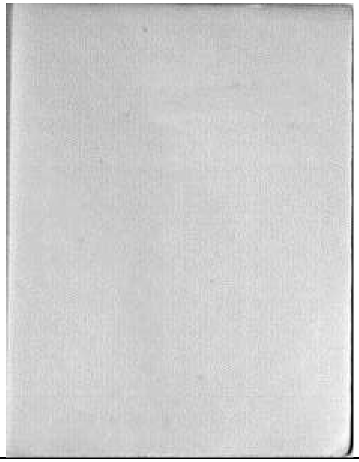	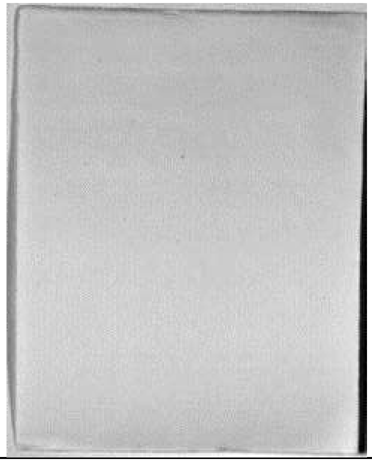	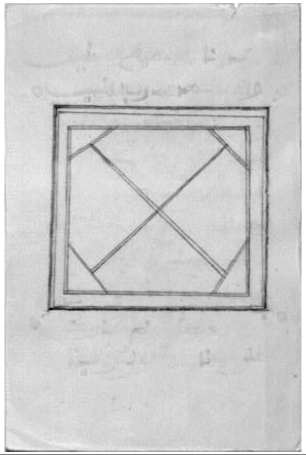
**SEM EM 24139**	**SEM EM 24140**	**SEM EM 24141**
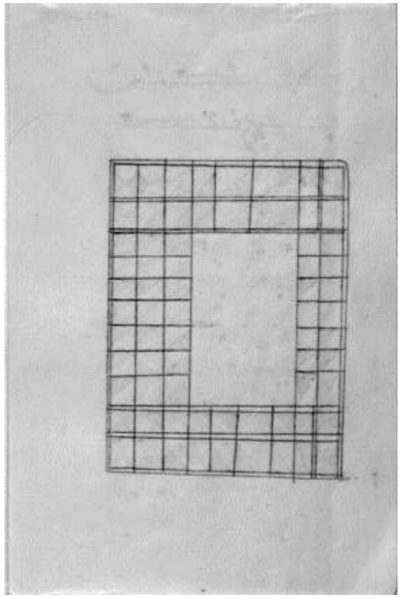	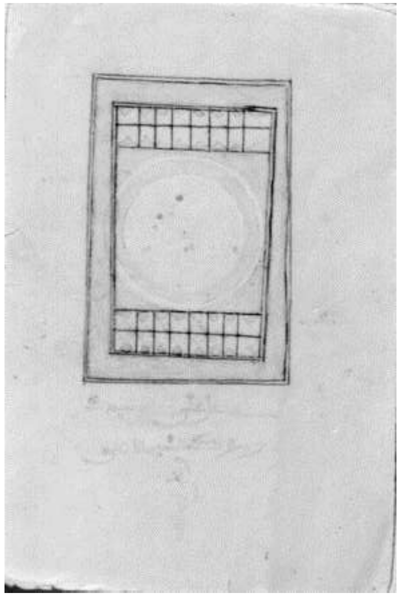	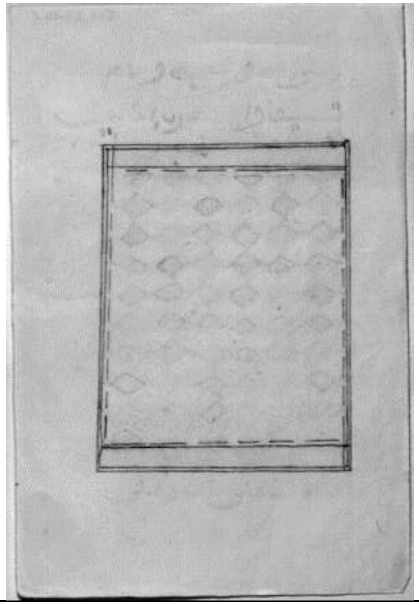
**SEM EM 24142**	**SEM EM 24143**	**SEM EM 24144**

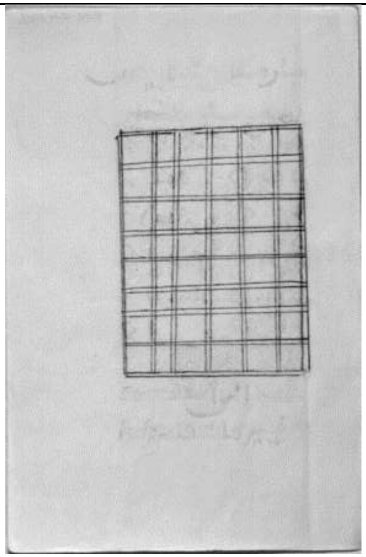	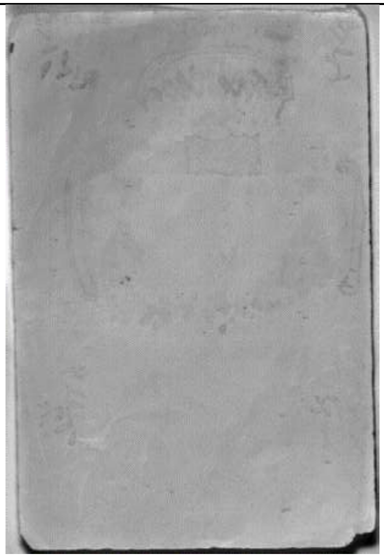	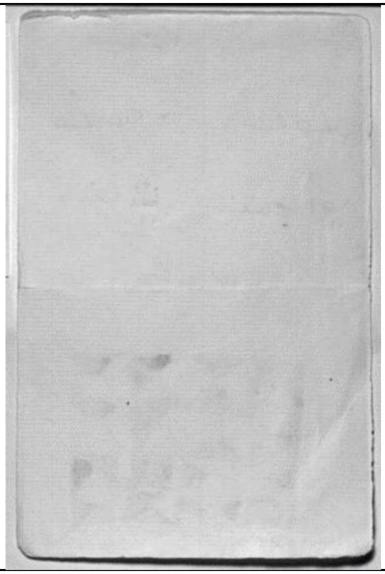
**SEM EM 24145 (part)**	**SEM EM 24146 (part)**	**SEM EM 24147 (part)**

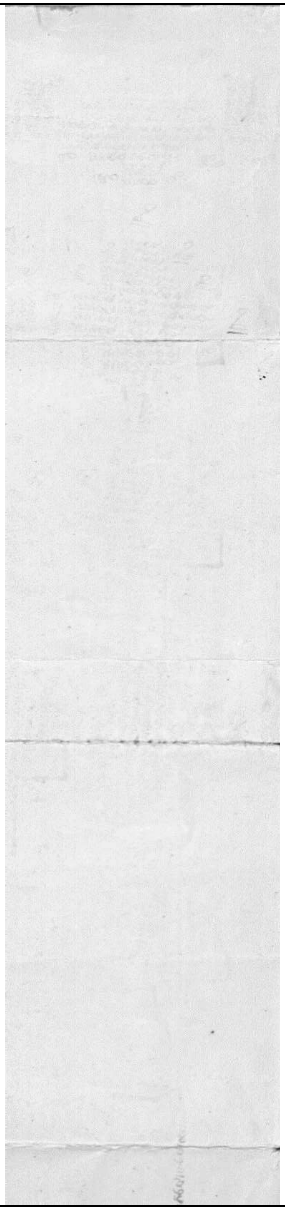

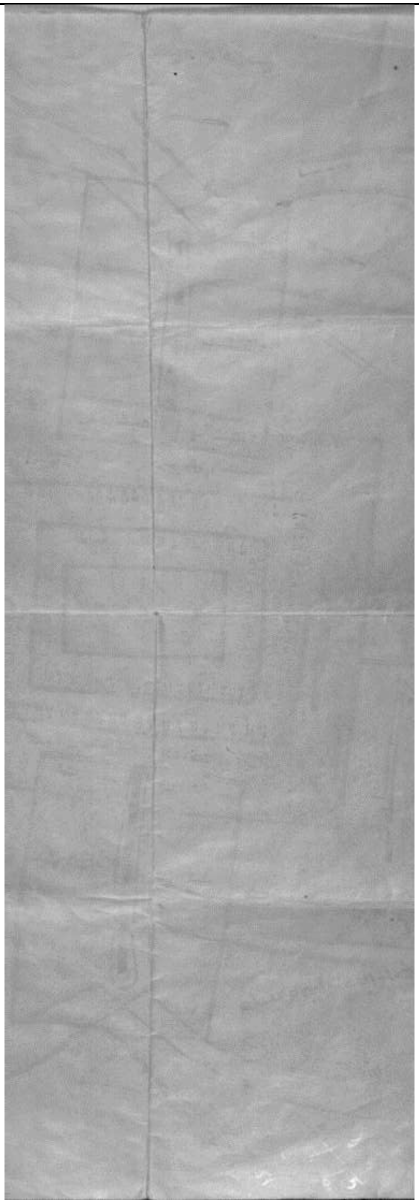

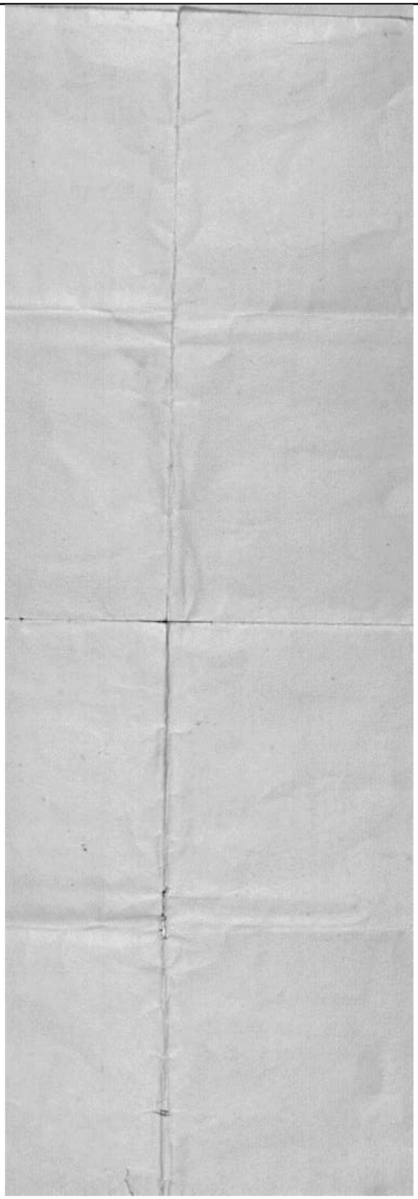

Table 6. Graphs of Raman Spectra of characteristic bands of black Iron Gall ink (IGI) of the selected EM objects

SEM EM 23134-10

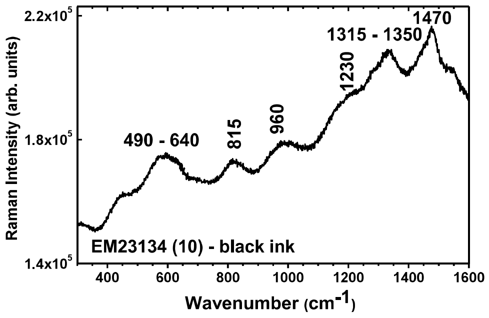

SEM EM 23134-25

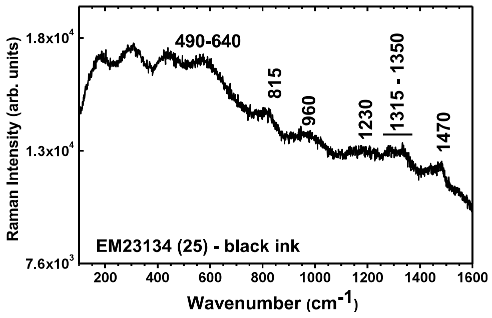

SEM EM 24141

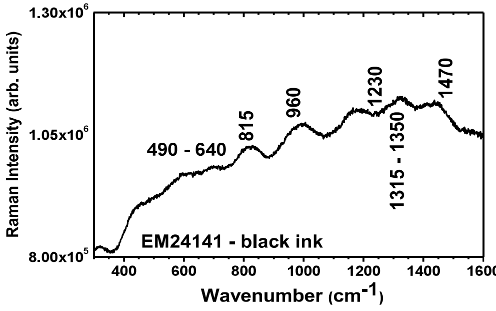

SEM EM 24143

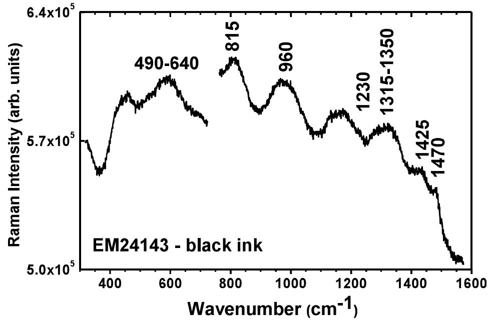

SEM EM 24144

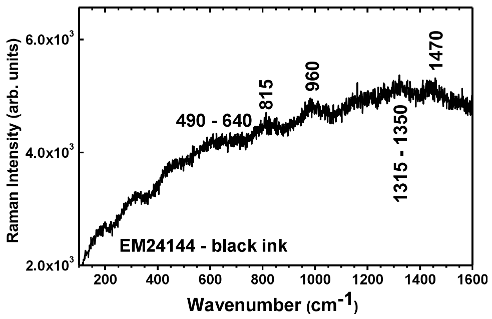

SEM EM 24145

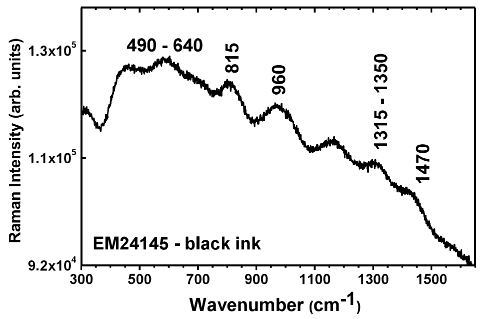

SEM EM 24146

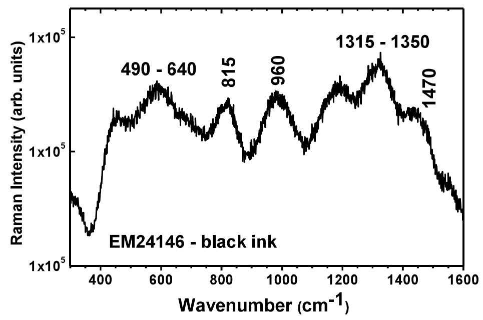

SEM EM 24147

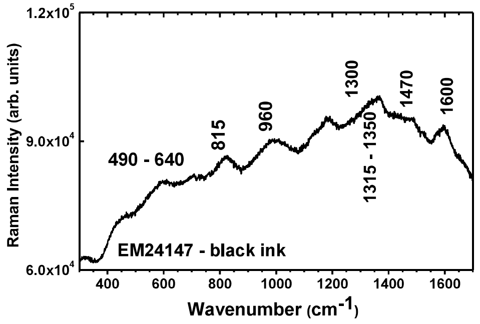

Ref Historical IGI sample

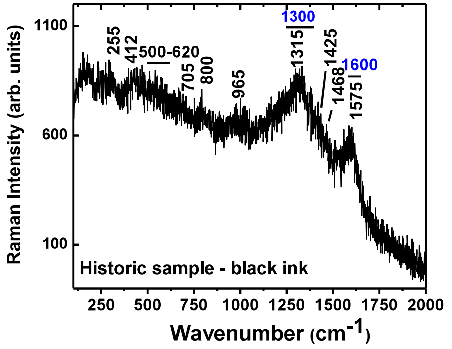

Table 7. Raman Spectra of characteristic bands of colored inks of the selected objects

SEM EM 23134-25 (Red ink)

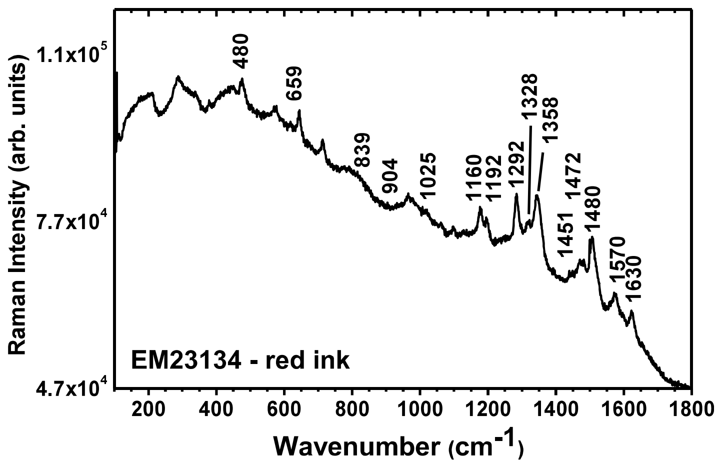

SEM EM 24141 (yellowish ink)

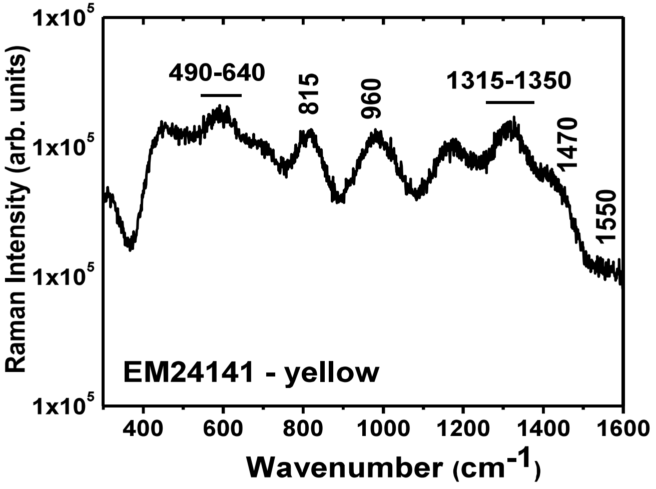

SEM EM 24145 (Red ink)

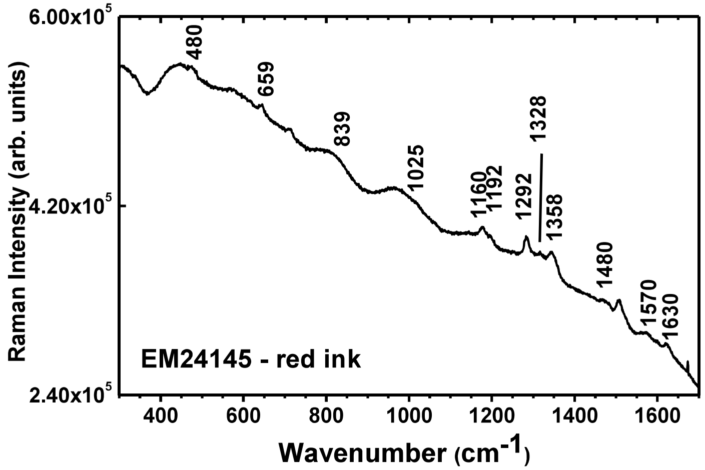

SEM EM 24141 (purple ink)

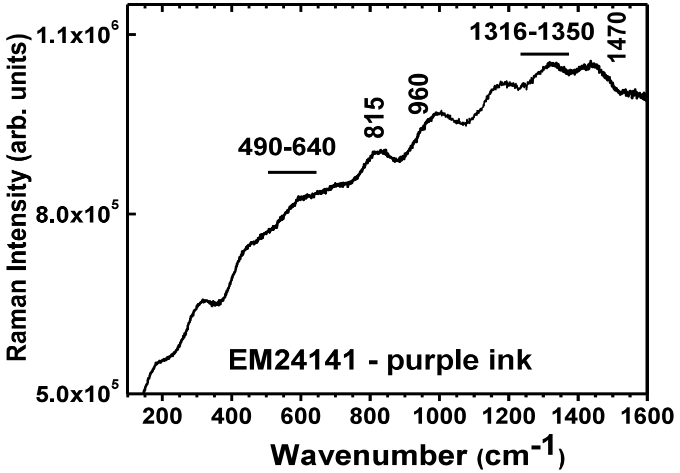

Table 8. LIF graphs of the selected objects

SEM EM 23134-10

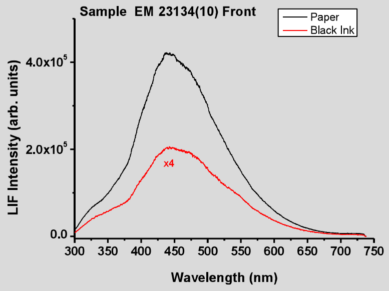

SEM EM 23134-25

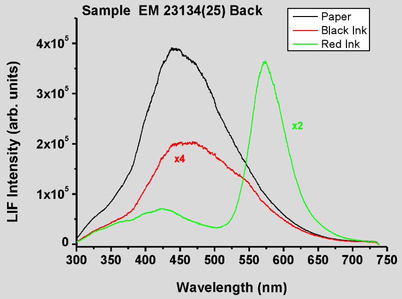

SEM EM 24140

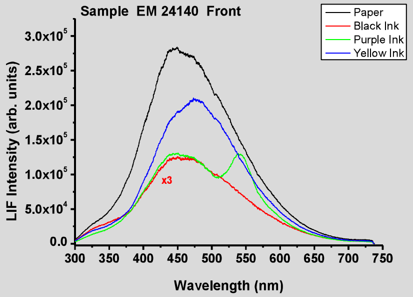

SEM EM 24141

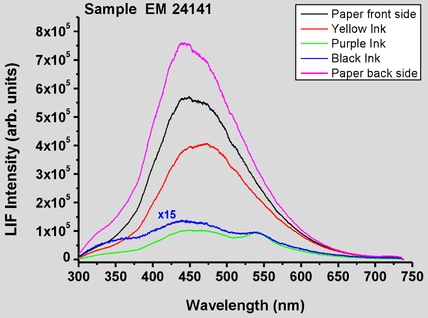

SEM EM 24143

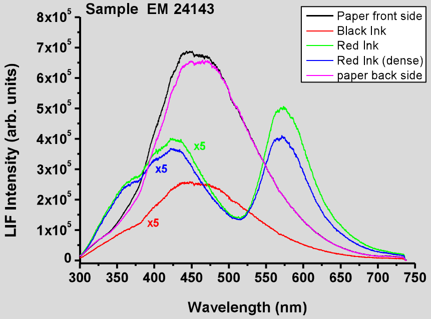

SEM EM 24144

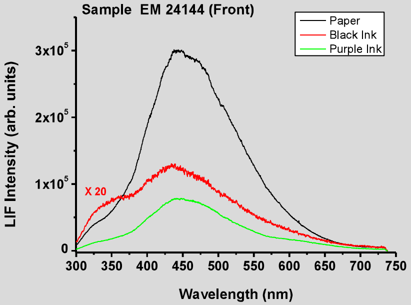

SEM EM 24145

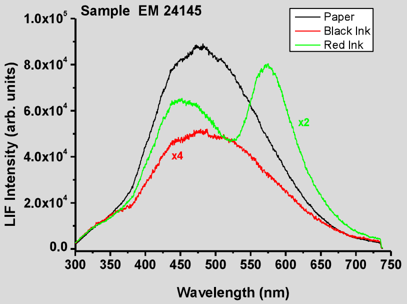

SEM EM 24146

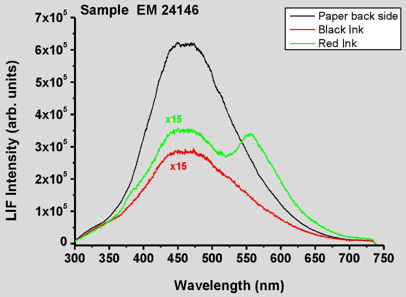

SEM EM 24147

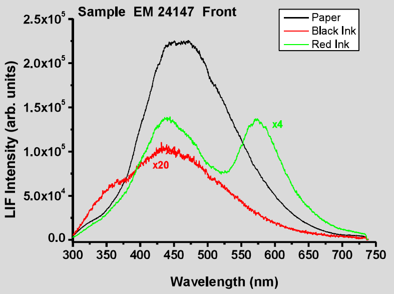

Table 9. LIBS graphs of the selected objects

SEM EM 23134-10

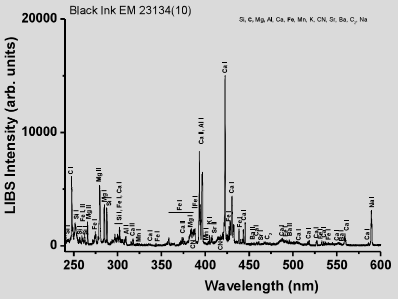

SEM EM 23134-25

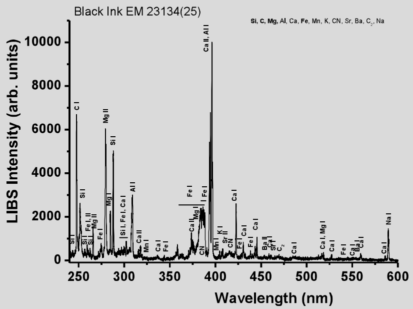

SEM EM 24143

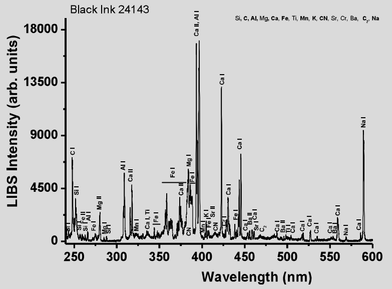

Reference historical sample of carbon-based ink

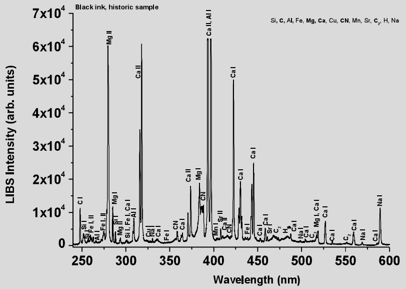

SEM EM 24143- red ink

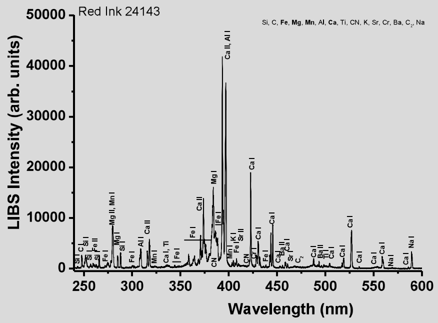

Reference historical sample of red ink

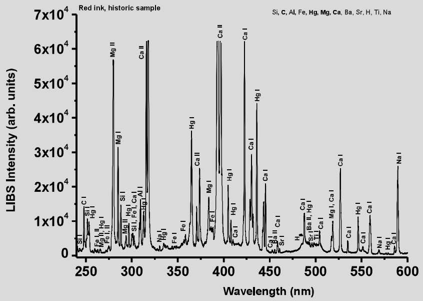

SEM EM 24143 – paper substrate

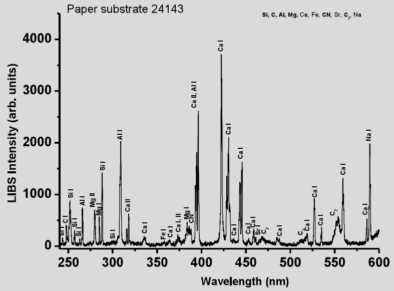

Reference historical sample of paper

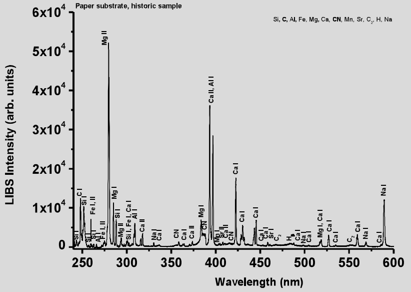

Reference model sample of paper

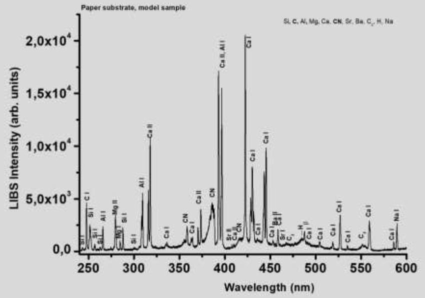

Reference model sample of IGI ink

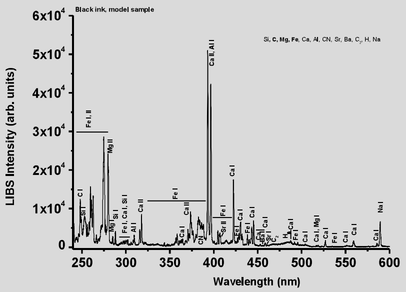

Table 10. The hyperspectral imaging system's acquisition parameters.

Camera	VNIR		SWIR	
Spectral Range (nm)	400-1000		980-2500	
Spatial Pixels	340 px		384 px	
Spectral Channels	260		288	
Bit Resolution	12 bits		12 bits	
Spectral Sampling	2.3 nm		5.6 nm	
Lens focal length	50 mm (F/8)	35 mm (F/8)	56 mm (F/2)	30 mm (F/2)
Exposure	125 ms	76.4 ms	7 ms	6.8 ms
Target Distance	610 mm	530 mm	580 mm	500 mm
Scan Speed	2.3 mm/s	4.6 mm/s	27 mm/s	44.4 mm/s
FOV/iFOV	137.2/0.37 mm	170.3/0.45 mm	95.3/0.31 mm	153.3/0.5 mm
Frame Rate (fps)	7.964	13.014	108.493	110.898

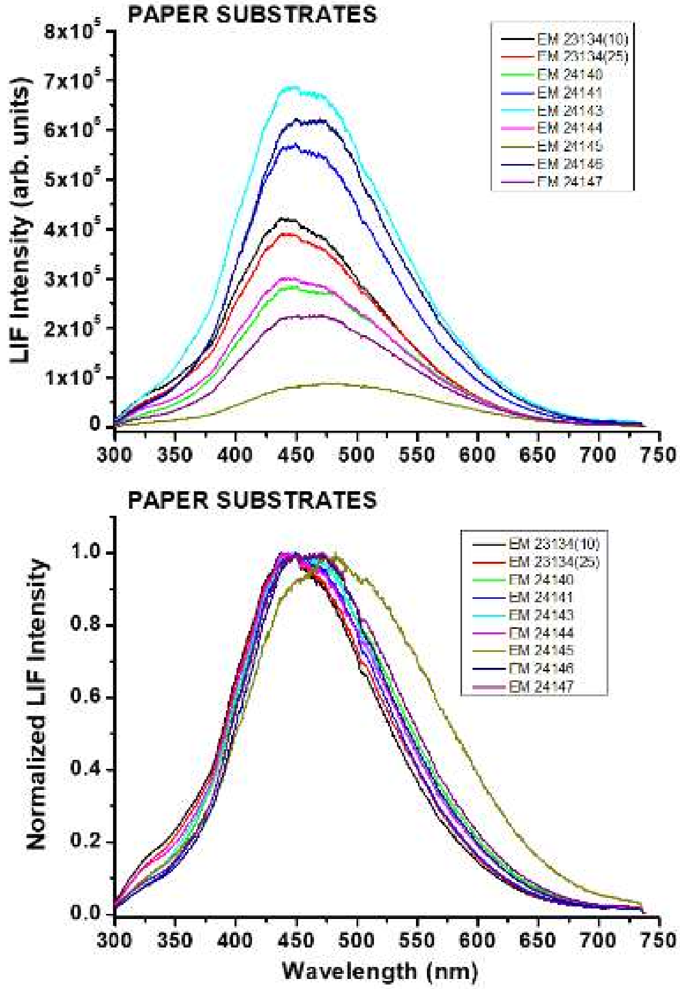

Figure 1: LIF spectra of paper substrates from different samples upon laser excitation at 266 nm (top is LIF spectra as collected using gate delay and width of 0 and 3 μs, respectively;

bottom: normalized LIF spectra for comparison, both LIF spectra correspond to the accumulation of 15 measurements).

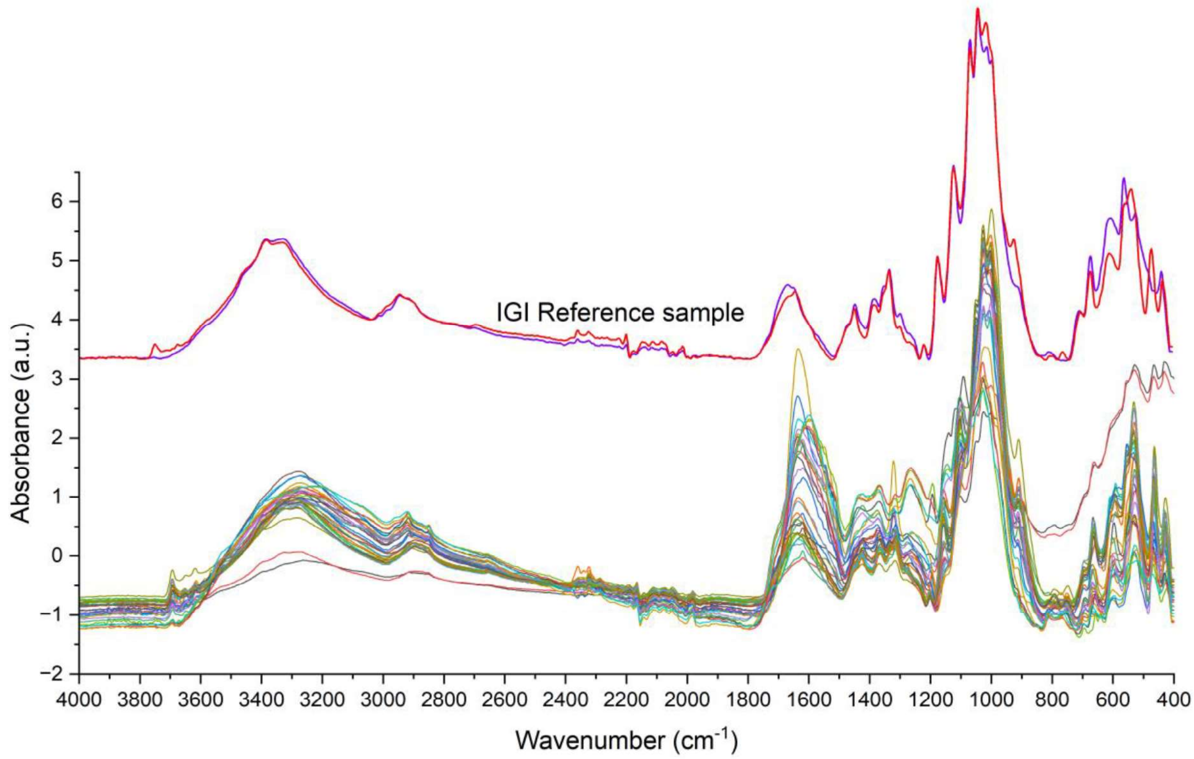

Figure 2. FTIR spectra of black inks collected from 12 EM objects, including samples from both drawings and text. The spectra show characteristic features associated with iron gall ink (IGI) components. For comparison, two IGI reference spectra from the historic HSLL collection are also included.
